# Aluminum Alloy Hot Stamping and Forming Technology: A Review

**DOI:** 10.3390/ma18081694

**Published:** 2025-04-08

**Authors:** Ruolin Wu, Wei Dai, Jiake Luo, Mengxin Li, Yuan Liu, Huanhuan Li

**Affiliations:** 1 Hubei Province Key Laboratory of Chemical Equipment Intensification and Intrinsic Safety, Wuhan Institute of Technology, Wuhan 430205, China; wruolin@wit.edu.cn (R.W.); daiwei2710@163.com (W.D.); jiake6516@163.com (J.L.); mengxin3260@163.com (M.L.); 2 Hubei Provincial Engineering Technology Research Center of Green Chemical Equipment, Wuhan Institute of Technology, Wuhan 430205, China; 3 School of Mechanical and Electrical Engineering, Wuhan Institute of Technology, Wuhan 430205, China

**Keywords:** HFQ^®^, pre-hardened hot forming, LT-HFQ^®^, heat deformation behavior, strength prediction model, heat treatment

## Abstract

Aluminum alloy hot stamping technology has quickly become a research hotspot for many scholars due to its ability to solve key challenges such as poor formability, large rebound, and low dimensional accuracy of aluminum alloy sheets at room temperature. This work systematically reviews the progress of Hot-Forming-Quenching (HFQ^®^) technology and its optimization processes. The effects of key forming parameters are summarized, including temperature, forming rate, friction, and crimping force on the forming properties of aluminum alloys. Additionally, an ontological model of thermal deformation behavior and damage evolution during hot forming is analyzed. A multifactorial strength prediction model, integrating grain size and reinforcement mechanisms, is highlighted for its ability to accurately predict post-forming yield strength. To address the limitations of HFQ^®^, optimization methods for solid solution and aging heat-treatment stages are categorized and evaluated, along with their advantages and disadvantages. Furthermore, the latest advancements in two innovative hot stamping processes (Low-Temperature Hot Form and Quench (LT-HFQ^®^) and pre-hardened hot forming (PHF)) are reviewed. LT-HFQ^®^ improves formability by pre-cooling the sheet while maintaining solution treatment, while PHF utilizes pre-hardened aluminum alloys, enabling brief heating, forming, and quenching to significantly reduce cycle time while ensuring component strength. Finally, by summarizing current technological progress and challenges, future directions for aluminum alloy hot stamping are outlined, including advancements in forming processes, material modeling, and optimization through multidisciplinary collaboration and artificial intelligence to drive further innovation.

## 1. Introduction

With the rapid growth in the automotive industry, the expansion of production scale and increasing emissions has intensified global energy consumption and environmental pressures. The emergence of new energy vehicles has reshaped the global automotive market, driving the industry toward energy-efficient and environmentally friendly transformations [1,2]. However, the limited range of electric vehicles remains a critical challenge. Studies have shown that for traditional fuel-powered vehicles, a 10% reduction in vehicle mass can improve fuel efficiency by 6–8% and reduce emissions by 4%. For electric vehicles, a 100 kg reduction in total mass can extend the range by approximately 15 km [3,4,5,6]. Consequently, vehicle lightweighting is widely recognized as the most direct and effective strategy for reducing energy consumption and emissions. Given that the body constitutes about 40% of a vehicle’s total mass, reducing its weight is essential for achieving lightweighting objectives. Lightweighting technology has become a key competitive factor for automakers and a major focus of innovation in automotive engineering [7]. In an era of increasing environmental awareness, advancing the automotive industry toward sustainability and eco-friendliness is vital for addressing global energy and environmental challenges.

**Figure 1:**
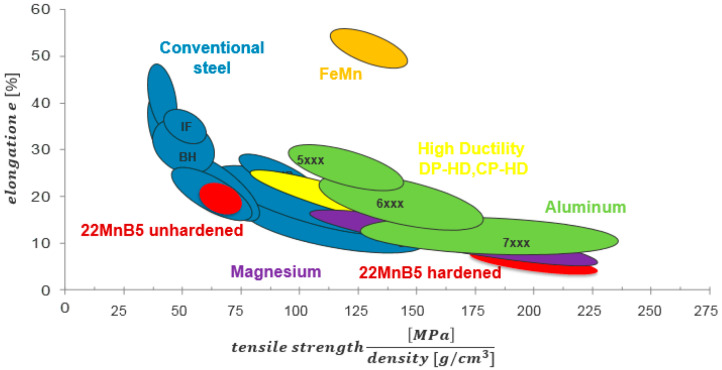
Lightweighting potential of metallic materials [15].

Automotive lightweighting technology primarily focuses on three areas: lightweight materials, advanced manufacturing processes, and structural optimization [8]. Numerous studies have been conducted to explore these aspects at the component level [9,10,11]. While advanced manufacturing processes such as laser welding, hydroforming, thermoforming, roll forming, and high-vacuum die casting have made significant progress in addressing material processing challenges, further improvements are needed in defect control, process optimization, and material adaptability. In terms of structural optimization, automotive body structures are predominantly composed of thin-walled components, which are limited by stiffness constraints. Consequently, achieving substantial lightweighting effects through structural optimization alone is challenging. Therefore, in addition to integrated component design, the use of lightweight materials with high strength-to-weight ratios has been demonstrated to offer significant potential for lightweighting. Currently, lightweight materials such as high-strength steels, aluminum alloys, and various composites are widely utilized in the automotive, aerospace, and electrical industries. High-strength aluminum alloys have become a key focus for global automotive manufacturers due to their exceptional properties, such as low density, high specific strength and stiffness, excellent impact resistance, strong corrosion resistance, and efficient heat dissipation [12,13]. These alloys also provide cost advantages over other materials. Compared to titanium, magnesium, and other light alloys [14], aluminum alloys demonstrate significant potential for further development. Notably, 7000 series aluminum alloys and press-hardened steels have been shown to offer considerable advantages in the automotive sector. The lightweight design potential of various metal materials is illustrated in Figure 1 [15,16]. The 5XXX series aluminum alloys are non-heat-treatable and are primarily strengthened through work hardening, as their solid solution compositions remain stable during heating. In contrast, the 2XXX, 6XXX, and 7XXX series are heat-treatable alloys, which can be strengthened via aging and precipitation hardening to enhance their mechanical properties. Among these, the 2XXX series alloys are particularly valued in the aerospace industry due to their exceptional strength-to-weight ratio. Automotive manufacturers in developed regions, including Europe and the United States, have widely adopted aluminum alloys in vehicle bodies [17,18,19,20]. Initially, their use was limited to body panels for sports cars, high-end sedans, and the white bodies of luxury vehicles such as BMW, Land Rover, and Audi [21,22]. Today, aluminum alloys used in automobile bodies are primarily classified into the 2XXX, 5XXX, 6XXX, and 7XXX series. The 2XXX, 5XXX, and 6XXX series are mainly used in structural, decorative, and heat dissipation systems, while the 7XXX series is employed in thermoformed structural components. Relevant data highlight the current applications of aluminum alloys in select vehicles [23,24,25,26,27]. Changan Automobile has successfully implemented an aluminum bonnet cover in models such as the CV11. Similarly, the 2017 Cadillac CT6 incorporates 11 different lightweight materials, with aluminum alloys accounting for over 57% of the structure. The vehicle also utilizes aerospace-grade manufacturing processes, significantly enhancing the body’s strength, performance, and efficiency, resulting in a lightweight yet robust design. The 2018 Audi A8 features aluminum in 58% of its body structure, employing cast, extruded, and sheet forms. Despite the widespread use of aluminum alloys in the automotive industry, their application is constrained by poor room-temperature formability and significant springback during cold stamping [28,29,30]. To address these limitations, researchers have developed superplastic-forming (SPF) and quick plastic-forming (QPF) technologies [31,32,33,34]. However, SPF and QPF require fine-grained materials and are costly. W-state stamping, conducted after solid solution quenching but before aging, suffers from low productivity. In contrast, hot stamping technology has gained widespread adoption in the automotive industry, particularly for producing body-in-white components such as A-pillars, B-pillars, bumpers, and rocker rails [35,36]. This process significantly improves the formability of aluminum alloys and effectively controls springback [37], establishing the way for a broader application of high-strength aluminum alloys in automotive structural components.

In recent years, the research concerning aluminum alloy hot stamping technology has mainly focused on the influence of forming parameters such as stamping speed and initial temperature on forming performance, as well as rheological properties and damage failure prediction based on intrinsic models. However, in industrial applications, challenges such as long hot stamping cycles and mismatched production rates have limited its widespread adoption. Consequently, exploring new technological approaches such as rapid solution treatment and rapid aging for the heat-treatment stages has become a key research focus. These advancements aim to enhance production efficiency and product quality, addressing current limitations and driving further innovation in the field. The emergence of new aluminum alloy hot stamping processes, such as LT-HFQ^®^ and PHF, represents a significant effort to reduce hot stamping cycle times. Research in this area has primarily focused on the core innovations of these processes, their practical application outcomes, and the analysis and evaluation of their technological merits. Despite extensive research, a systematic review combining the optimization of traditional hot stamping processes with these new approaches has yet to be reported. Therefore, this paper reviews the development of aluminum alloy hot stamping technology; summarizes recent advancements in traditional hot stamping, including process optimization, performance enhancement, thermal deformation behavior, and yield strength prediction models; and analyzes the innovative core and application potential of new hot stamping processes. Building on existing research, the paper also discusses future prospects for aluminum alloy hot stamping technology, offering development directions and recommendations grounded in industrial needs. This work aims to provide a valuable reference for subsequent research and development in the field.

## 2. HFQ^®^ Process Study

**Figure 2:**
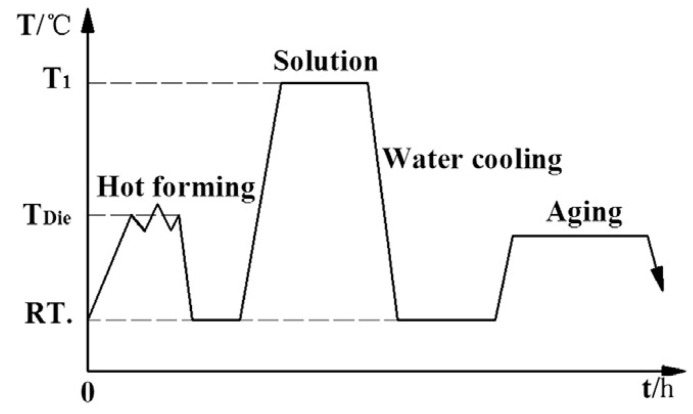
Traditional aluminum alloy hot-forming process [40].

**Figure 3:**
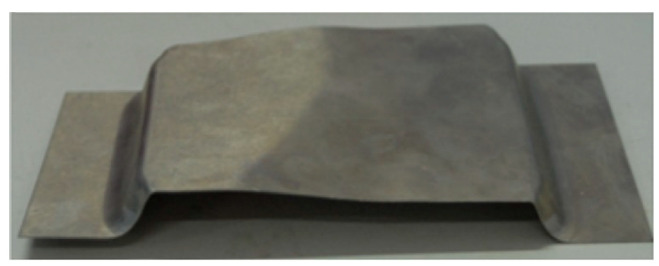
Parts under traditional aluminum alloy hot-forming process [40].

In the traditional aluminum alloy hot-forming process, forming is typically performed before heat treatment. After forming, the parts undergo heat treatment to enhance their mechanical properties. This treatment consists of three key steps (Figure 2). Solution heat treatment (SHT): The material is held at a high temperature long enough to dissolve all alloying elements into a solid solution, forming a single-phase structure. Quenching: The material is rapidly cooled from the SHT temperature to room temperature to “freeze” the microstructure as a supersaturated solid solution (SSSS). Aging: Age hardening allows controlled decomposition of the SSSS, forming fine precipitates that enhance strength, corrosion resistance, and fatigue life [38]. However, post-forming heat treatment can lead to poor formability, springback, and distortion [39]. For example, Fan et al. [40] observed part distortion in conventional hot-forming studies of 6A02 aluminum alloy (Figure 3).

**Figure 4:**
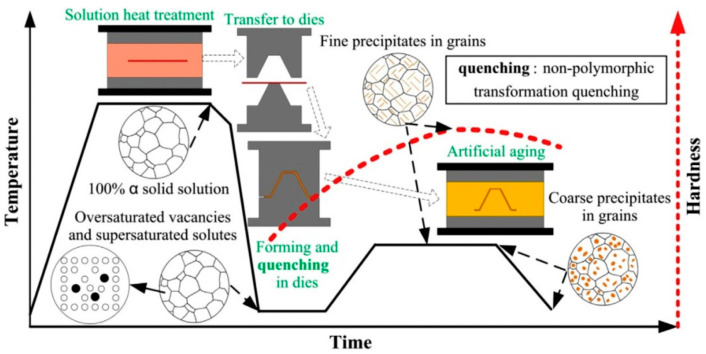
Hot stamping and forming process and organization evolution of heat-treatable reinforced aluminum alloys [2].

For this reason, Lin [41] from Imperial College, UK, first proposed an innovative integrated process combining heat treatment and hot forming for aluminum alloys, known as the HFQ^®^ process. This method was initially tested for its feasibility on 2XXX, 6XXX, and 7XXX series aluminum alloys [42,43]. As shown in Figure 4, the fully solution-treated aluminum alloy sheet is rapidly transferred to a water-cooled die and immediately formed. The die remains closed to complete in-die quenching, after which the part undergoes aging treatment to achieve its final mechanical properties [44]. The deformation mechanism in this process primarily involves age hardening, where second-phase particles precipitate from the supersaturated solid solution as the alloy transitions from a single-phase to a two-phase region. This precipitation sequence follows the typical behavior observed in hot-stamped 7XXX-series aluminum alloys. For instance [45]:

$$\begin{array}{c} Solid\;solution\to GP\;zones\to metastable\eta^{\prime }\\ \to stable\;\eta (MgZn_{2}) \end{array}$$

As shown in Figure 4, heating the sheet to solution temperature and holding for a prescribed duration produces a supersaturated solid solution through polymorphic transition quenching (non-isotropic transition), where solute atoms and vacancies become trapped in a metastable state. During artificial aging, this supersaturated solution first forms coherent Guinier–Preston (GP) zones—ordered solute clusters that strengthen the alloy. These subsequently transform into semi-coherent η′ precipitates (typically 1–10 nm in size) [46] before finally evolving into stable η-phase precipitates during over-aging [47]. The HFQ^®^ process ensures that the aluminum alloy sheet rapidly forms a supersaturated solid solution during quenching, reducing material hardness and flow stress, thereby improving formability during stamping. In-die quenching also enhances the strength and dimensional accuracy of the sheet while minimizing springback. Subsequent artificial aging significantly increases the strength of the parts and addresses issues such as poor room-temperature plasticity and shape distortion during heat treatment [48,49]. Additionally, the HFQ^®^ process not only improves forming performance compared to traditional stamping but also enables the creation of complex, one-piece stamped parts without disassembly. By using high-strength aluminum alloy sheets formed in a single piece, the need for internal reinforcements is reduced, part assembly is simplified, and multiple component connections are eliminated, all while reducing the number of molds required.

The HFQ^®^ process integrates forming and heat treatment of aluminum alloy sheets. For different series of aluminum alloys, the HFQ^®^ process requires tailored forming parameters and heat-treatment regimes, which collectively influence the forming and mechanical properties of the final parts. Extensive simulation is essential to determine the optimal process parameters. Additionally, controlling the cycle time of hot stamping is critical to maximizing the industrial value and efficiency of this advanced forming technology.

### 2.1. Hot Deformation Behavior of HFQ^®^

The forming process is a critical aspect of aluminum alloy hot stamping, directly influencing the accuracy and quality of the final part [50]. Consequently, factors such as forming speed, temperature, and friction significantly impact the forming properties of the sheet.

**Figure 5:**
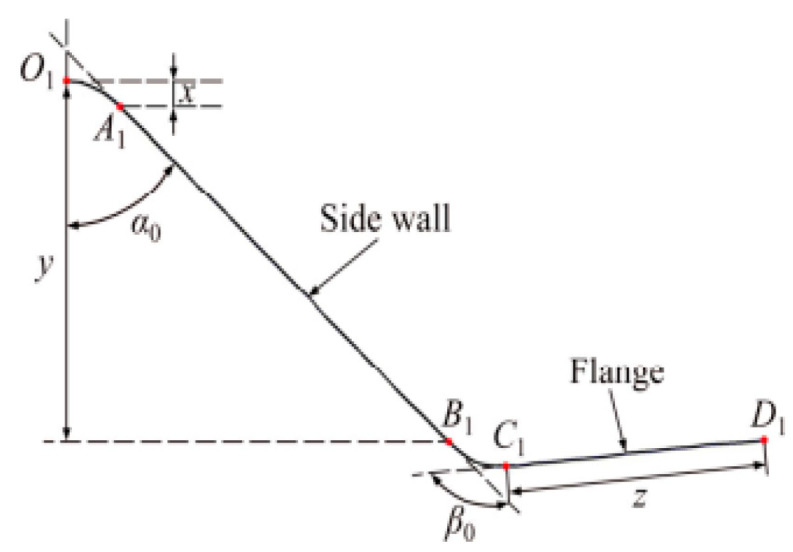
Measurement of angles *α*_0_ and *β*_0_ after springback [52].

**Figure 6:**
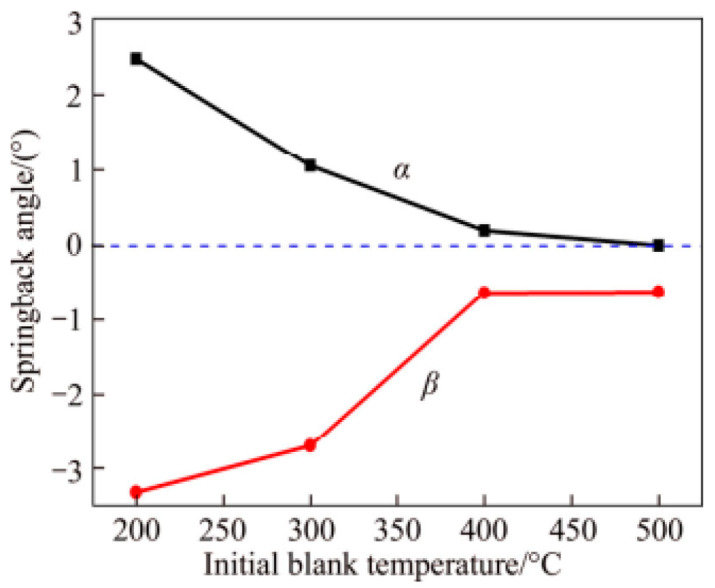
Experimental springback angle changing with initial blank temperature [52].

**Figure 7:**
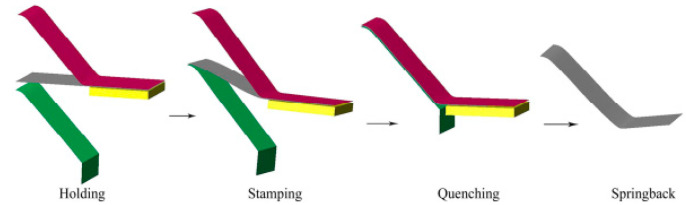
Four simulation stages for predicting springback of v-shaped stamped parts [52].

Wang et al. [51] analyzed the springback of AA5754 using experiments and finite element simulations, concluding that springback increases with the tangential stress gradient. Ma et al. [52] experimentally investigated the springback behavior of 6016 aluminum alloy during hot stamping using a V-die test. The measurement method is shown in Figure 5, As shown in Figure 6, the springback angle exhibits strong temperature dependence, decreasing significantly within the 200–400 °C range but showing minimal variation between 400 and 500 °C. The researchers validated these experimental findings through finite element modeling (Figure 7), with the simulation results demonstrating good agreement with experimental measurements (Table 1). Furthermore, springback is significantly influenced by mold parameters [53,54]. An increase in the angular radius of the grinding tool leads to a higher springback angle. Although the HFQ^®^ process significantly reduces springback, slight springback persists after unloading the thermoformed parts. Zhou et al. [55] analyzed the springback behavior of 7075 aluminum alloy thermoforming at 25–400 °C by experiment and finite element simulation, and effectively predicted the springback angle change.

**Figure 8:**
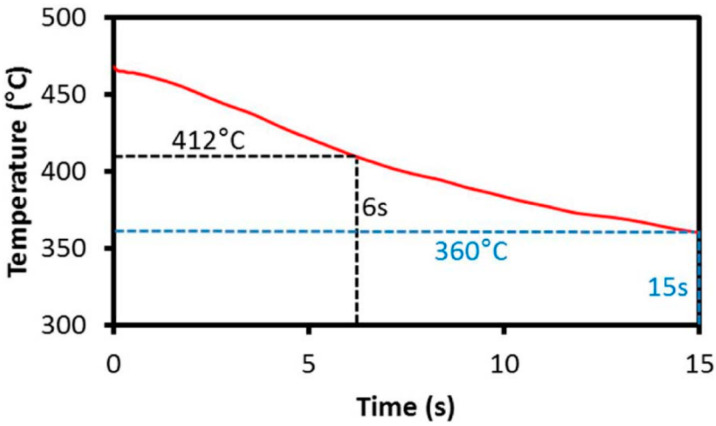
Temperature drop of AA7075 during transfer between furnace and press [58].

**Figure 9:**
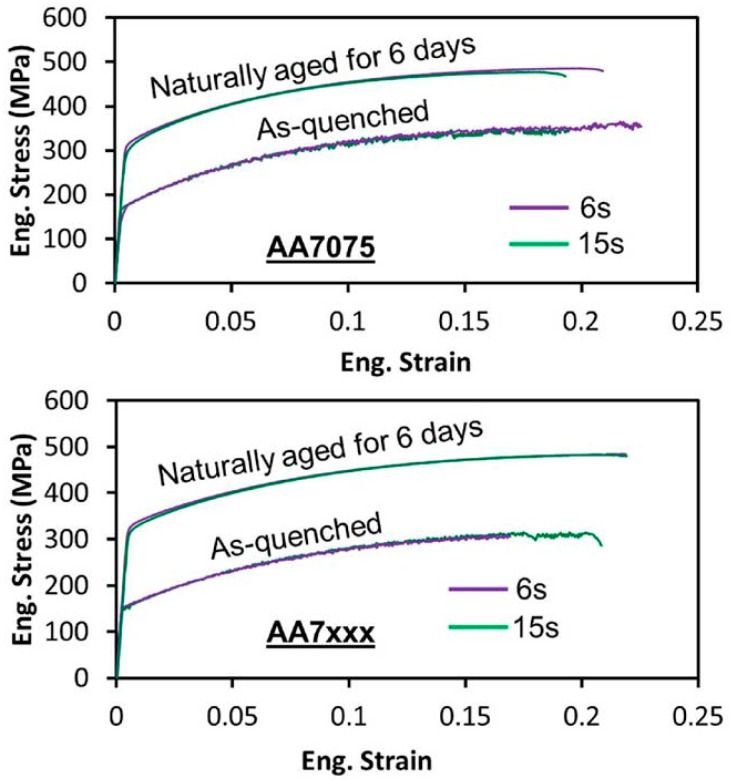
Engineering stress–strain curves for different transfer times [58].

Ma et al. [56] studied the hot stamping properties and high-temperature deep drawing behavior of AA6111 aluminum alloy sheets. Their simulations revealed that larger crimping forces and higher friction coefficients increased material elongation but reduced the minimum thickness. Punch velocity is a major factor in thickness deviation because high punch speeds keep the heat transferred to the tool during the forming process to a minimum. Relatively uniform temperatures help to homogenize deformation and reduce thickness deviation. Xiao et al. [57] investigated the forming performance of 7075 aluminum alloy through simulations of hot deformation and high-temperature deep-drawing tests. They found that forming performance improved at lower temperatures, with reduced crimping force and friction coefficient. When the billet was heated to around 400 °C and well lubricated, forming properties improved significantly, with optimal performance achieved at a stamping speed of 50 mm/s. However, non-uniform deformation can easily lead to surface cracks in the workpiece. Liu, Yong et al. [2] carried out tensile tests on 6061 aluminum alloy at a temperature of 350–500 °C and a strain rate of 0.01–1 s^−1^ and simulated the established Cowper–Symonds eigenstructural equations and the experimental COF values, explaining the mechanism of crack generation during hot stamping of 6061 aluminum alloys, which is due to unfavorable friction caused by inhomogeneous cooling and the large temperature difference between the sidewalls and rounded corners. Lubrication not only reduces friction and promotes metal flow but also mitigates temperature differences. However, high sliding speeds can lead to localized oxide generation on the wear surface, reducing the coefficient of friction. During the transfer of the sheet to the forming die, brief cooling occurs due to air contact. Omer et al. [58] investigated the effect of transfer time (from the furnace to the forming die) on the hardening response of AA7075 and a new 7XXX aluminum alloy (AA7XXX). Their results (Figure 8) showed that while air exposure during transfer causes slight cooling, longer transfer times do not significantly affect hardening. However, AA7075 exhibited some loss of elongation at a transfer time of 15 s (Figure 9), whereas AA7XXX demonstrated lower sensitivity to quenching rate and transfer time. Zheng et al. [59] further studied how blank transfer influences material deformation during extrusion and its aging behavior, ultimately affecting the final material properties.

**Figure 10:**
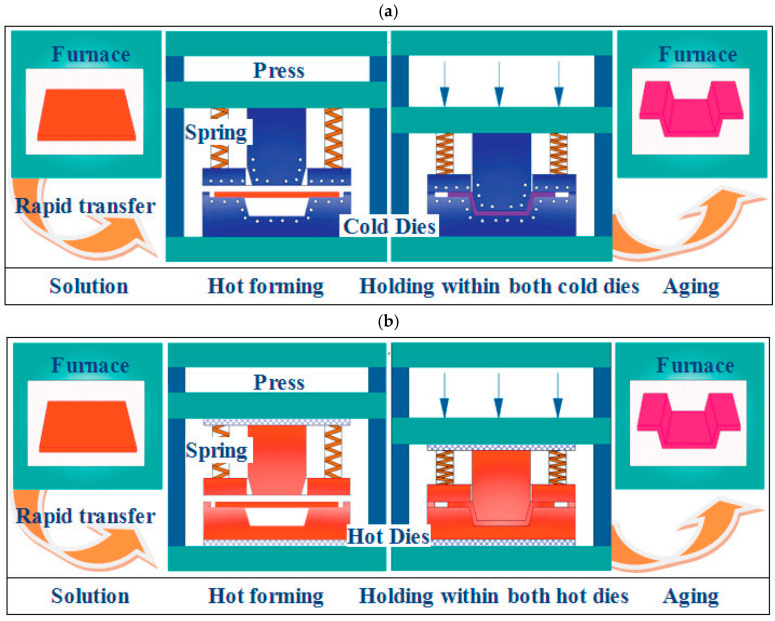

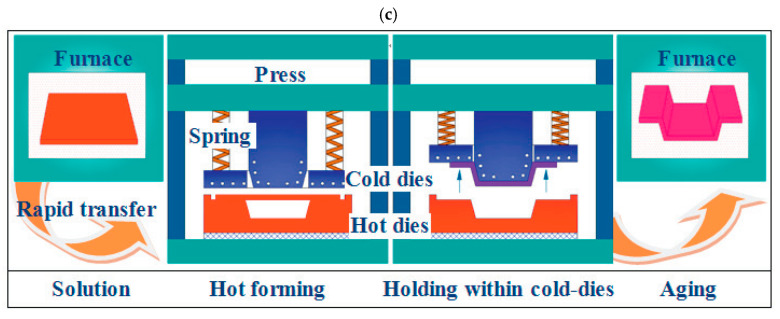
Schematic diagram of the integrated thermoforming–quenching process using (**a**) double cold die, (**b**) double hot die, and (**c**) hot and cold die [62].

**Figure 11:**
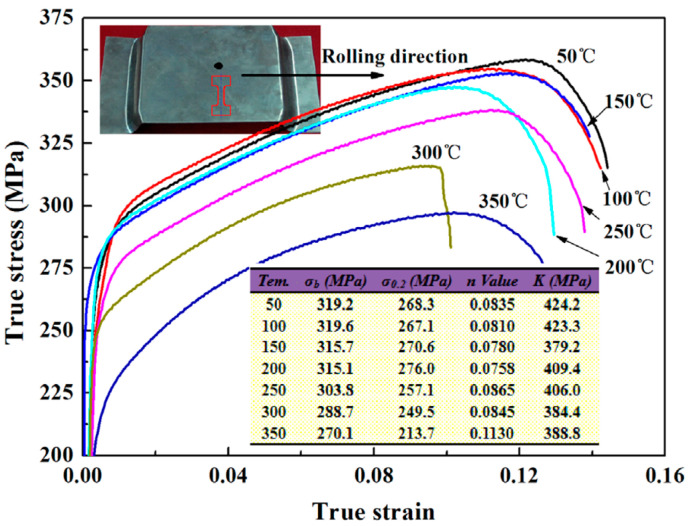
Mechanical properties of the parts formed at different temperatures of the forming dies in the integrated process [40].

**Figure 12:**
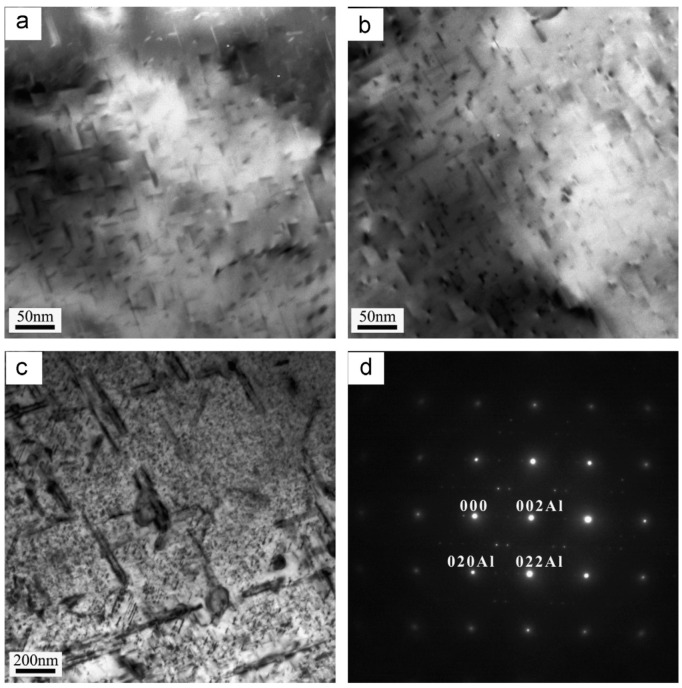
TEM images of precipitates at different temperatures of the forming dies: (**a**) 50 °C, (**b**) 200 °C and (**c**,**d**) 350 °C [40].

When the sheet is fed into the mold, the temperature difference between the mold and the sheet significantly affects the forming process. Fan et al. [40] systematically investigated the integrated hot forming-quenching process of 6A02 aluminum alloy sheets across mold temperatures ranging from 50 °C to 350 °C (Figure 10b). Their analysis of the formed parts’ mechanical properties (Figure 11) revealed a gradual decrease in both hardness and strength with increasing forming temperature, with a particularly pronounced reduction occurring at 250 °C. Microstructural examination (Figure 12a,b) showed abundant, uniformly distributed needle-like precipitates within the aluminum matrix at 50 °C and 200 °C. These observations align with the established precipitation sequence for 6XXX-series alloys [60,61]: Al(SSSS)-{atomic clusters (Mg, Si)}-{formation of GP zones}-{β″ precipitates}-{β′ precipitates}-{β Mg_2_Si precipitates}. When the mold temperature is kept below 250 °C, rapid cooling promotes the formation of fine, high-density β″ precipitates (10–50 nm) uniformly dispersed in the aluminum matrix. These coherent, needle-like precipitates create strong lattice strain fields that effectively hinder dislocation motion, resulting in superior mechanical properties, including tensile strengths exceeding 300 MPa. However, when the mold temperature rises above 250 °C (Figure 12c), slower cooling rates lead to precipitate coarsening, transforming the β″ phase into larger (150–300 nm) rod-like β’ precipitates with reduced coherency. This microstructural change significantly weakens the strengthening effect due to diminished strain fields and the segregation of β’ precipitates and α-Al intermetallic compounds at grain boundaries, which further deteriorates grain boundary cohesion. Although some grain coarsening occurs at elevated temperatures, the primary factor governing material strength remains the evolution of precipitates rather than grain size. Thus, to maximize strengthening, the mold temperature must be maintained below 250 °C to ensure a sufficient population of fine β″ precipitates. Experimental measurements under these conditions demonstrate a Vickers hardness of 100.6 HV, along with tensile and yield strengths of 303.8 MPa and 257.1 MPa, respectively. Yuan et al. [62] proposed a hot–cold mold thermoforming-quenching process (Figure 10), where the lower mold is heated, and the upper die is cooled. This method slows the temperature drop of the sheet while maintaining high strength. Subsequent research by Fan, Xiaobo et al. [63] on the integrated hot forming–quenching process revealed that in dual-temperature die forming (combining cold and hot dies), the strength of the formed parts decreased significantly when the upper die temperature reached 250 °C, while the lower die temperature could be increased to 450 °C without similar strength reduction. Their study confirmed the precipitation sequence for Al-Cu-Mg alloys as follows [64,65,66]:

$$SSSS\to co-clusters\to S^{\prime \prime }/GPB2\to S$$

**Figure 13:**
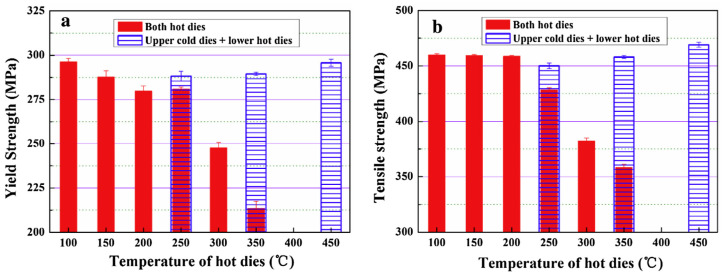
Relationship of mechanical properties on temperature of hot dies: (**a**) yield strength, (**b**) tensile strength [63].

The cold–hot die combination offers distinct advantages in the forming process: heating the lower die prevents rapid temperature loss in the sheet, maintaining optimal formability, while the upper die’s rapid cooling ensures sufficient quenching rates to suppress coarse S-phase formation. This controlled thermal management promotes the precipitation of fine, uniformly distributed strengthening phases (50–100 nm) during subsequent aging, resulting in excellent mechanical properties. In contrast, the double hot die configuration suffers from significantly reduced cooling capacity due to excessive mold temperatures, which promotes the formation of coarse S-phase precipitates (750–1000 nm) during quenching and consequently diminishes material strength. The optimal mechanical properties were achieved at a mold temperature of 250 °C, with the material exhibiting a yield strength of 295.7 MPa and tensile strength of 469.2 MPa, as demonstrated in Figure 13. In addition, they further explored the effect of molding temperature (25–500 °C) on material properties based on Al-Mg-Si alloys, and found that peak hardness occurs at 200 °C and 500 °C, while good formability and strength can be obtained, which can be used as the applicable molding temperature [67]. Recent studies have also demonstrated that reducing the die gap improves the dimensional accuracy of Al-Cu-Mg alloy sheets in the hot forming–quenching integrated process. A smaller die clearance enhances heat transfer efficiency during quenching, enabling better control of temperature distribution and ensuring the alloy achieves peak age strengthening while maintaining high strength [68].

**Figure 14:**
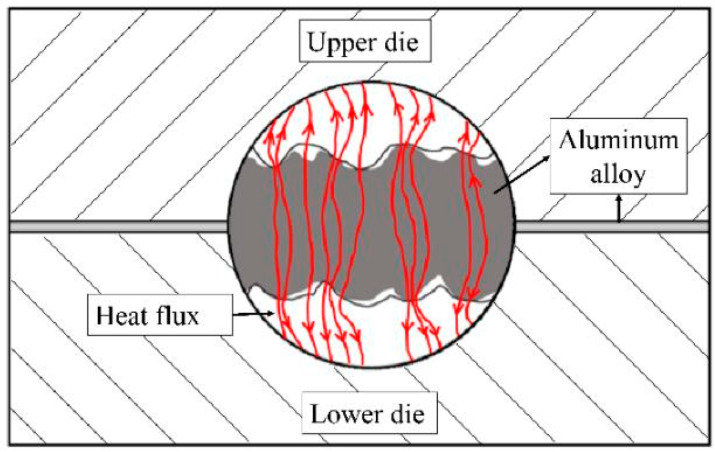
Microscopic morphology of the interface between aluminum alloy and mold [69].

In the HFQ thermoforming process, the heat transfer phenomenon occurs in the contact between the heat-deformed aluminum alloy and the low-temperature mold; due to the radiation and convection heat loss to the surrounding air being negligible, the heat transfer from the alloy to the mold is mainly the heat conduction within the metal, due to the rough surface of the alloy and the mold, and the cavity is filled with air or other media, which results in the existence of an air gap between the alloy and the mold and hinders the heat transfer, as shown in Figure 6. Figure 6 demonstrates the microscopic morphology of the interface between the aluminum alloy and the die, hence the existence of thermal resistance. The condition of the contact surface between the alloy and the mold affects the heat transfer, different factors including contact pressure, surface roughness, surface lubrication, etc., affect the condition of the contact surface, such as the actual contact area, gap thermal conductivity, etc., which ultimately affects the thermal resistance, and thus the interfacial heat transfer coefficient (IHTC). The IHTC reflects the heat transfer efficiency between the mold and the alloy sheet. Ying et al. [69] investigated the transient heat transfer behavior of high-strength 7075-T6 alloy during the HFQ^®^ process using a cylindrical mold model, with the microscopic morphology of the aluminum alloy–mold interface illustrated in Figure 14. Xiao et al. [70] observed that the IHTC of AA7075 increases with contact pressure, and lubrication significantly enhances heat transfer efficiency. However, when contact pressure is very low, in-die quenching performance deteriorates. Zhou et al. [71] developed a finite element model for aluminum alloy hot stamping, using a door impact side beam as an example. They combined a response surface model, sampling techniques, and a multiobjective genetic algorithm to optimize the process.

The rapid cooling of the sheet within the die creates significant temperature and deformation gradients, causing the material to undergo complex strain and temperature changes. These conditions activate deformation mechanisms such as work hardening, dynamic recovery, and dynamic recrystallization, involving intricate thermomechanical behaviors. Zhou et al. [72] investigated the hot tensile deformation behavior of Al-Zn-Mg-Cu alloys and classified the true stress–strain curves into four stages: elasticity, homogeneous deformation, diffusion necking, and local necking. Their results indicate that flow stress decreases with increasing temperature or decreasing strain rate, while elongation at fracture increases with temperature. Xiao et al. [73] analyzed the flow behavior and microstructural evolution of AA7075 using uniaxial hot tensile tests. EBSD results revealed that grain size progressively refines with increasing deformation, higher temperatures, and lower strain rates. Qiang et al. [74] identified dynamic recovery as the primary softening mechanism during isothermal hot compression of 7A04 aluminum alloy. Microstructural analysis confirmed the thermal deformation behavior at temperatures of 350–480 °C and strain rates of 0.002 s^−1^ to 20 s^−1^. Wang et al. [75] investigated the formability of AA2024 in the temperature range of 350–493 °C through tensile tests. They found that plasticity peaked near 450 °C, but ductility sharply decreased beyond this temperature. They also observed that plasticity increased with the tangential stress gradient, and a similar trend was noted for the ductility of AA5754. Additionally, forming temperature significantly influenced springback behavior.

To optimize hot forming process parameters, the stress–strain relationship, microstructure evolution, and damage behavior of aluminum alloys under high-temperature conditions are analyzed through ontological relationships. These relationships connect process parameters to the material’s macroscopic deformation behaviors, offering insights for process improvement. Commonly used constitutive models for thermoplastic deformation are categorized into empirical models, physically based models, and artificial neural network models [76]. Among these, empirical models are widely used to simulate metal forming at high temperatures and strain rates. Typical examples include the Arrhenius model [77,78,79], the Johnson–Cook model [80,81,82], and the Fields–Backofen (FB) model [81]. However, purely physically based constitutive models often struggle to accurately capture deformation characteristics due to their limited ability to describe microstructural evolution. Therefore, it is essential to develop physically based constitutive models that incorporate material properties, dislocation dynamics, and thermal activation energy. Representative models include the Zerilli–Armstrong (ZA) model [83], the dynamic recrystallization (DRX) model [84], and the cellular automata (CA) model [85]. 7075 is a typical high-strength 7XXX series aluminum alloy. In studies of its hot deformation constitutive relationship, the Arrhenius-type constitutive model is the most commonly used, as it is well suited for predicting peak flow stress. Wang et al. [86] measured the stress–strain relationship of 7075 aluminum alloy at temperatures of 350–500 °C and strain rates of 10^−3^–10^−2^ s^−1^ using high-temperature tensile tests on double-roll cast material. They fitted the data to an Arrhenius-type constitutive model. The modified Zerilli–Armstrong (ZA) model can also be used to predict flow stresses in materials at high temperatures [87,88,89]. Yi et al. [90] studied the thermal compressive deformation behavior of 7050 high-strength aluminum alloy within the temperature range of 250–450 °C and strain rates of 10^−2^–10 s^−1^, constructing an Arrhenius constitutive equation incorporating Zener-Hollomon parameters. Lin et al. [91] accurately predicted the high-temperature stress–strain curve of 2124 aluminum alloy by refining the parameters of the Arrhenius constitutive equation.

Empirical constitutive models can provide relatively accurate predictions of the hot deformation behavior of heat-treatable aluminum alloys based on experimental data. However, their credibility is limited because they cannot fully explain the physical mechanisms governing material deformation. In contrast, physically based constitutive models can precisely characterize deformation behavior by incorporating microscopic mechanisms such as dislocation density, grain growth, texture evolution, and cavity formation [92]. For example, Lin et al. [93] defined a normalized dislocation density ($\bar{\rho }$) to establish a dislocation density evolution model, incorporating mechanisms such as thermal deformation, recrystallization, and static/dynamic recovery:

(1) $$\dot{\bar{\rho }}={\left(\frac{d}{d_{0}}\right)}^{\gamma_{4}}\left(1-\bar{\rho }\right){\dot{\varepsilon }}^{p}-c_{1}{\bar{\rho }}^{c_{2}}-\left(\frac{c_{3}}{1-S}\right)\dot{S}$$

where *d* is the average grain diameter; *S* is the recrystallization volume fraction; $d_{0}$, $\gamma_{d}$, $c_{1},\;c_{2}$, and $c_{3}$ are material constants; $\bar{\rho }$ = 1 − $\rho_{i}$/ρ, where $\rho_{i}$ is the initial dislocation density and ρ is the dislocation density during deformation. The value of $\bar{\rho }$ ranges from 0 to 1. The recrystallization volume fraction, *S*, is defined by an empirical formula, and its evolution can be described by Equation (2) [93]:

(2) $$\dot{S}=Q_{0}\left[x\bar{\rho }-{\bar{\rho }}_{c}\left(1-S\right)\right]{\left(1-S\right)}^{N_{q}}$$

where $Q_{0}$ and $N_{q}$ are constants; ${\bar{\rho }}_{c}$ is the critical value of normalized dislocation density. The parameter x describes the onset of recrystallization and is defined as $\dot{x}=A_{0}(1-x)\bar{\rho }$, where $A_{0}$ is a material constant. The recrystallization volume fraction cyclically varies between 0 and 1.

The unified viscoplastic constitutive model can accurately describe the thermoplastic deformation behavior of metals by incorporating microscopic deformation mechanisms such as dislocation density, grain size, and cavity growth damage evolution. A typical set of unified viscoplastic constitutive equations is as follows [94]:

(3) $$\left\{\begin{array}{l} {\dot{\varepsilon }}_{P}={\left[\frac{\frac{\sigma }{1-f_{d}}-R-H}{K}\right]}^{1}\\ \dot{R}=0.5J{\bar{\rho }}^{-0.5\dot{\bar{\rho }}}\\ \dot{\bar{\rho }}=N\left(1-\bar{\rho }\right)\left|{\dot{\varepsilon }}_{P}\right|-L{\bar{\rho }}^{M}\\ {\dot{f}}_{d}=of_{d}^{S}{\dot{\varepsilon }}_{P}^{W}+P{\dot{\varepsilon }}_{P}^{U}\cos h\left(Q_{\varepsilon }\right)\\ \sigma =E\left(1-f_{d}\right)\left(\varepsilon -\varepsilon_{P}\right) \end{array}\right.$$

(4) $$\bar{\rho }=\left(\rho -\rho_{0}\right)/\left(\rho_{\max}-\rho_{0}\right)$$

where ${\dot{\varepsilon }}_{P}$ is the plastic strain rate; $f_{d}$ is the damage region fraction; R is the hardening variable; H is the yield strength, $\bar{\rho }$ (0 $\leq \bar{\rho }\leq$ 1) is the normalized dislocation density; ρ  is the dislocation density; $\rho_{max}$ is the maximum dislocation density; $\rho_{0}$ is the initial dislocation density; $\varepsilon_{P}$ is the plastic strain; *E* is the elastic modulus; *I*, *J*, *K*, *L*, *M*, *N*, *O*, *P*, *Q*, *S*, *W*, and *U* are material constants, and these constants are temperature and strain-rate dependent.

**Figure 15:**
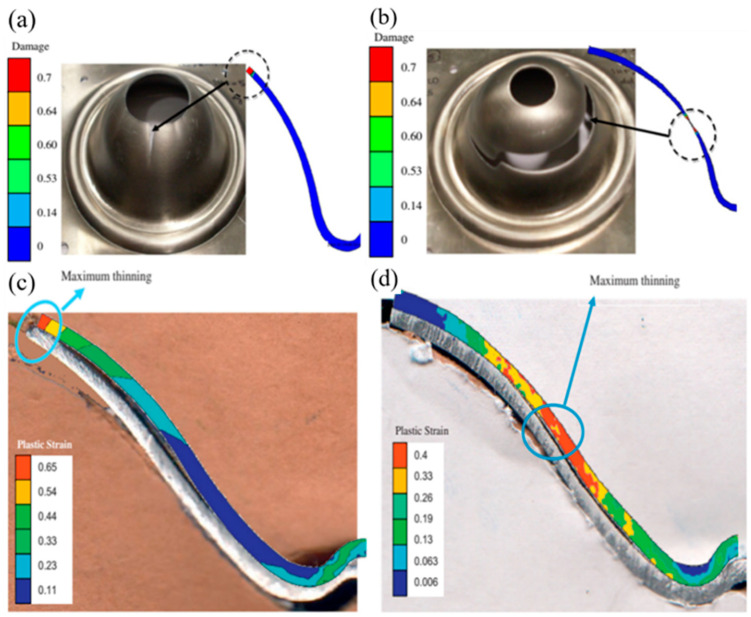
Experimental results and finite element formability simulations showing damage parameter contours of AA6082 plate deformed at 470 ± 10 °C. (**a**) Fast forming rate failure due to radial tearing of the center hole under 42 mm punch. (**b**) Slow forming rate failure due to circumferential tearing at mid-height position under 42 mm punch. (**c**) Comparison of finite element-simulated deformation profiles (including plastic strain profiles) with experimental deformed part cross-sections for fast forming rate under 32 mm punch and (**d**) slow forming rate under 32 mm punch [94].

**Figure 16:**
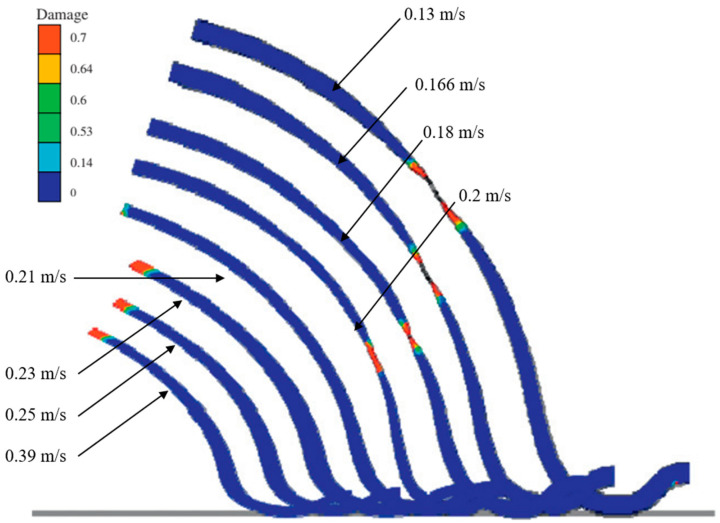
Failure characteristics and maximum thinning prediction of deformed cups at different forming rates [94].

**Figure 17:**
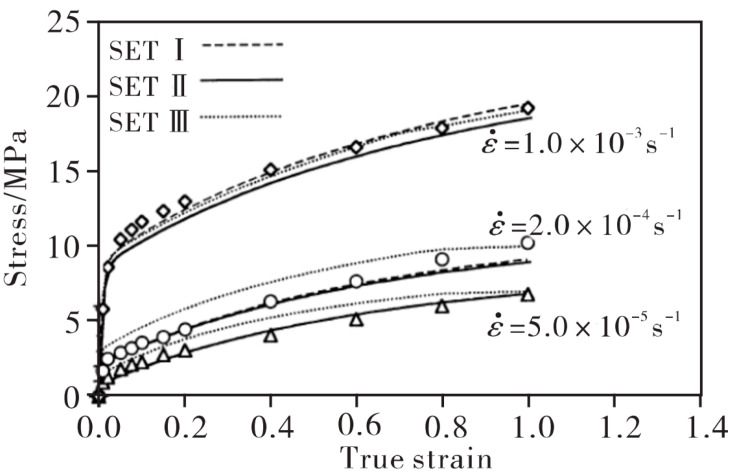
Predictions of stress–strain relationships versus experimental data [98].

Mohamed et al. [94] investigated the damage mechanisms of AA6082 alloy during high-temperature forming through hemispherical stamping experiments. As illustrated in Figure 15, their study revealed that the failure mode in hot stamping is governed by a complex interaction between forming rate and thermal distribution. Under low-speed forming conditions, prolonged contact between the blank and punch causes substantial cooling in the central region, creating a pronounced temperature gradient. This thermal gradient concentrates strain in the hotter, more ductile cup wall area, ultimately resulting in circumferential necking and fracture. Conversely, high-speed forming minimizes contact duration, preserving elevated temperatures and ductility in the central region. In this case, geometric stress concentrations (particularly at features like center hole edges) become the dominant factor, leading to radial necking and ductile tearing. These findings demonstrate a synergistic relationship between thermal gradients and part geometry in determining failure behavior, where the forming rate modulates the relative influence of each factor. Lower speeds emphasize temperature-dependent effects through contact cooling, while higher speeds shift dominance to geometric stress concentration effects. Subsequently, physical scalar relationship eigenequations containing internal state variables were developed at 450 °C and strain rate 1 s^−1^ to predict the failure characteristics and formability of AA6082 over a specific range of strain rates and temperatures, and applied to hot stamping simulations. Figure 15 illustrates the effects of different punch speeds on damage parameters and deformation cross-sections, comparing experimental and simulation results. This verification confirms the model’s ability to accurately predict the viscoplastic flow and damage behavior of AA6082. Figure 16 further demonstrates the failure characteristics and maximum thinning predictions of deformed cups at various forming rates. Although classical forming limit diagrams (FLDs) can describe necking failures under constant temperature and strain rate conditions, they are not directly applicable to predicting forming limits in hot stamping processes, where temperatures and strain rates vary dynamically. To address this, theories based on continuum damage mechanics (CDM) have been developed to predict damage processes and failures in various metal forming processes. Mohamed et al. [95] conducted formability tests aligned with CDM modeling, providing finite element analysis (FEA) material modeling tools for accurate failure prediction. Fakir et al. [96] evaluated the failure behavior of AA5754 aluminum alloy through uniaxial tensile and formability tests. They calibrated a viscoplastic constitutive model to predict stress–strain relationships and forming limit curves (FLDs) during deformation. This model integrates dislocation density hardening constitutive equations with the Hosford yield criterion to describe the anisotropic behavior of sheet metal and introduces a physically based damage model for predicting crack formation. Lin et al. [97] proposed a microdamage evolution model based on the primary deformation mechanisms of superplastic materials. They applied a unified viscoplastic damage constitutive equation to simulate the deformation behavior of Al-Zn-Mg and Al7475 alloys at 515 °C across their full strain-rate range and service life. Building on this, Lin [98] developed a series of viscoplastic constitutive equations incorporating a grain growth model, a hyperbolic sine law material model, and a power-law superplastic material model. The predicted stress–strain relationships were validated against experimental results, as shown in Figure 17. Furthermore, Lin et al. [99] established a new viscoplastic constitutive equation based on plane stress continuum damage mechanics (CDM), calibrated using tensile test data of AA5754 within a temperature range of 350–550 °C and strain rates of 0.1, 1.0, and 10 s^−1^. This model enables accurate predictions of forming limit curves (FLCs) for sheet materials under various hot stamping conditions. However, this equation involves a large number of parameters that require optimization and determination through experimental data, making parameter simplification a critical step to enhance the model’s practicality. Bai et al. [100] further developed the CDM-based AA5754 intrinsic equation for FLC experiments at 200–300 °C and 20–300 mm/s forming rates. By comparing the predictions of the finite element model with the results of dome-forming experiments, they found that the simulated crack locations and principal strain distributions closely matched the experimental values. This verification confirms the model’s accuracy in simulating the viscoplastic flow and damage accumulation of AA5754 until failure. The study also highlights the CDM model’s potential for predicting the forming performance of AA5754 in various thermoforming processes.

### 2.2. HFQ^®^ Strength Prediction Model for Formed Components

In aluminum alloy hot stamping and forming, the relationship between deformation state, aging system, and mechanical properties can be determined experimentally. However, the continuous expansion of high-strength aluminum alloy series results in variations in deformation and aging conditions, necessitating extensive testing. This approach is not only time-consuming and labor-intensive but also increases production costs. Therefore, developing strength prediction models to describe and predict the evolution of alloy mechanical properties is of significant scientific and practical value. These models serve as a crucial tool for optimizing the strengthening of aluminum alloys. In the field of heat-treatment research, numerous scholars have established various strengthening models based on fundamental strengthening mechanisms.

**Figure 18:**
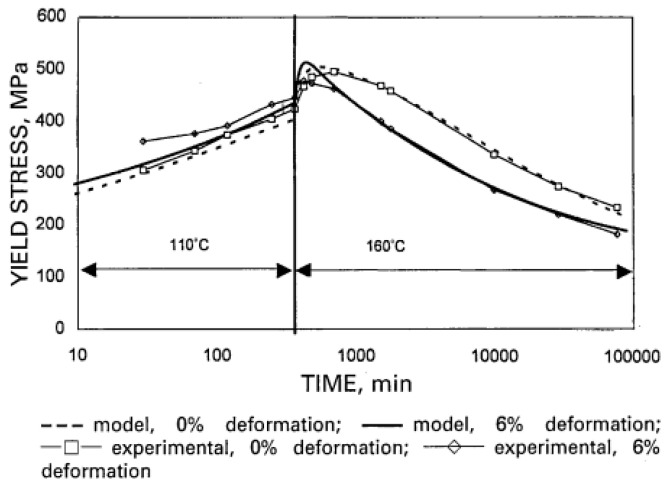
Comparison of model results with experimental results [103].

Shercliff and Ashby [101] established a pioneering model in 1990 to describe the age-hardening behavior of 2000-series aluminum alloys and 6000-series aluminum alloys. Their approach combined precipitation kinetics with microstructure–property relationships to systematically predict alloy yield strength. While this framework successfully explains the strengthening mechanisms in these alloy systems, the more complex precipitation behavior of 7000-series aluminum alloys necessitates additional model development to fully capture their aging response characteristics. Guyot et al. [102] conducted a comprehensive investigation of the age-hardening mechanisms in Al-Zn-Mg-Cu alloys, focusing on the temperature range for uniform η′ precipitation. Their study systematically correlated precipitation kinetics with mechanical properties and electrical conductivity, establishing quantitative relationships between microstructural evolution and mechanical performance. These findings provide fundamental insights into the age-hardening behavior of these alloys. For 7000-series aluminum alloys, optimal properties are typically achieved through a two-stage artificial aging process. This treatment enables the attainment of high strength while preserving adequate stress corrosion resistance, addressing the characteristic strength–corrosion trade-off in these alloys. In a two-step age-hardening study of 7475 aluminum alloy, Poole et al. [103] proposed a strength prediction model based on internal state variables, aiming at describing the effect of deformation on the aging kinetics. By introducing the contribution of precipitation strengthening and work hardening, the model also takes into account the evolution of the precipitate spacing and treats the non-isothermal aging process by the concept of “kinetic strength”. The model results are compared with the experimental measurements, as shown in Figure 18. The model predictions are in good agreement with the experimental results, especially near the peak strength and during the over-aging period, but there is a deviation from the experimental results during the initial aging stage, which the authors believe is related to the variation in the integral number of the precipitates and the choice of the contribution law to the flow stresses.

**Figure 19:**
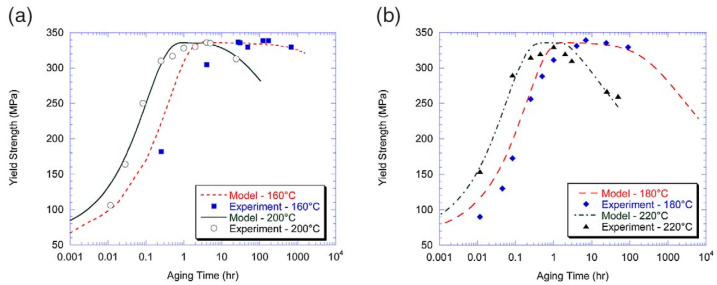
Comparison of the experimental and modeling results for the yield strength of solution-treated material using the strong obstacle assumption: (**a**) aging at 160 °C and 200 °C, (**b**) aging at 180 °C and 220 °C [106].

**Figure 20:**
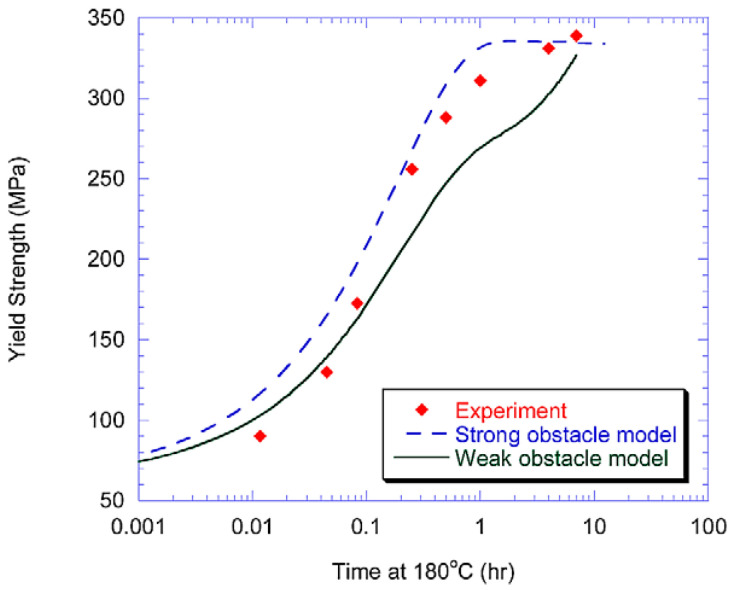
Comparison of the experimental and modeling results for yield strength during aging at 180 °C using both weak and strong obstacle assumptions [106].

In addition, after solid solution and quenching of the alloy, quenching stresses are relieved by plastic deformation in addition to multistep aging. Deschamps et al. [104,105] conducted systematic investigations into predeformation effects on aging behavior through integrated experimental and modeling approaches. Their work revealed that introduced dislocations substantially modify precipitation kinetics by altering the nucleation, growth, and coarsening behavior of strengthening phases. Notably, they identified a competitive mechanism between homogeneous precipitation and heterogeneous dislocation-assisted precipitation, with the dominant pathway being strongly influenced by heating rate. Esmaeili et al. [106] developed a yield strength prediction model for AA6111, modeling the contribution of precipitation hardening to yield strength for both strong and weak barrier scenarios. Their results suggest that dislocation–barrier interactions evolve as precipitates transition from weak to strong barriers depending on aging temperature. Using the strong-barrier model with the evolution of a single microstructural variable, they successfully predicted the yield strength of AA6111 during isothermal aging at 160–220 °C. As shown in Figure 19, the model predictions show good agreement with experimental data across the studied temperature range during aging. However, some discrepancies appear during the initial aging stages at 180 °C and 200 °C, as well as in under-aged conditions at 160 °C. Further analysis using the weak barrier model for the 180 °C aging case demonstrates improved predictive capability during early-stage aging (Figure 20).

**Figure 21:**
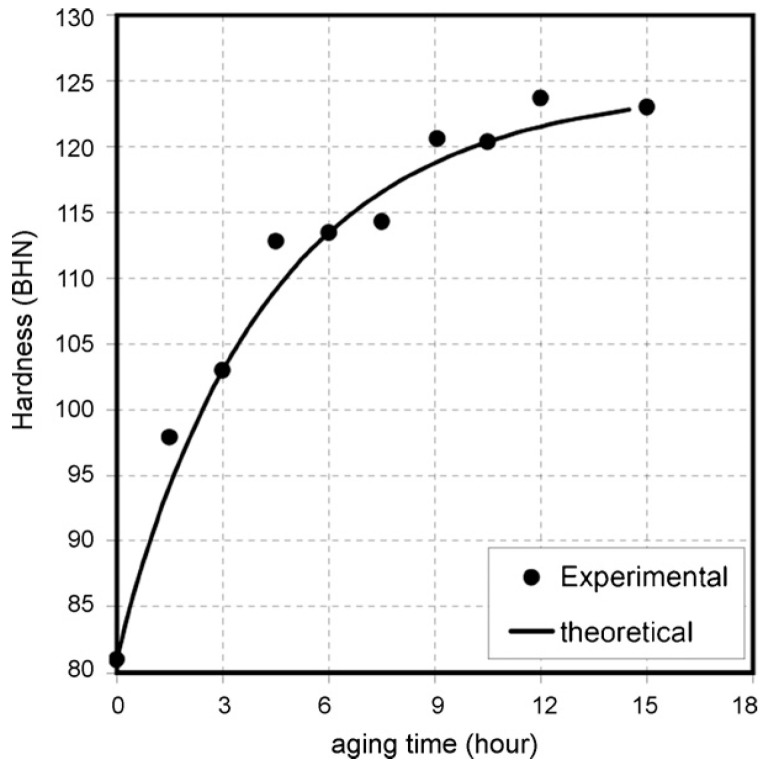
Comparison of theoretical and experimental results on precipitation hardening of Al-Mg-Si-Cu alloys at 190 °C [108].

Weakley-Bollin et al. [107] developed an innovative approach for simulating the age-hardening behavior of Al-Si-Cu cast alloys by correlating key microstructural parameters, such as {100} plate morphology, with hardening responses. Their work established a yield strength model for 319-type aluminum alloys (W319) capable of predicting mechanical properties based on copper concentration, aging temperature, and aging time. Eivani et al. [108] systematically investigated precipitation hardening kinetics in Al-Mg-Si-Cu alloys across the 125–225 °C temperature range. Their study derived predictive formulae for hardness evolution, identifying an optimal aging window of 15–18 h at 180–190 °C for achieving peak hardness. As demonstrated in Figure 21, the model predictions show excellent agreement with experimental measurements, validating the proposed relationships between processing parameters and mechanical properties. Hosseini-Benhangi et al. [109] employed an improved modeling approach, assuming that precipitates act as weak or strong barriers to dislocation motion. They combined this with a yield strength model for AA6111 alloy to derive a precipitation strengthening formula, enabling accurate prediction of yield strength evolution during artificial aging.

**Figure 22:**
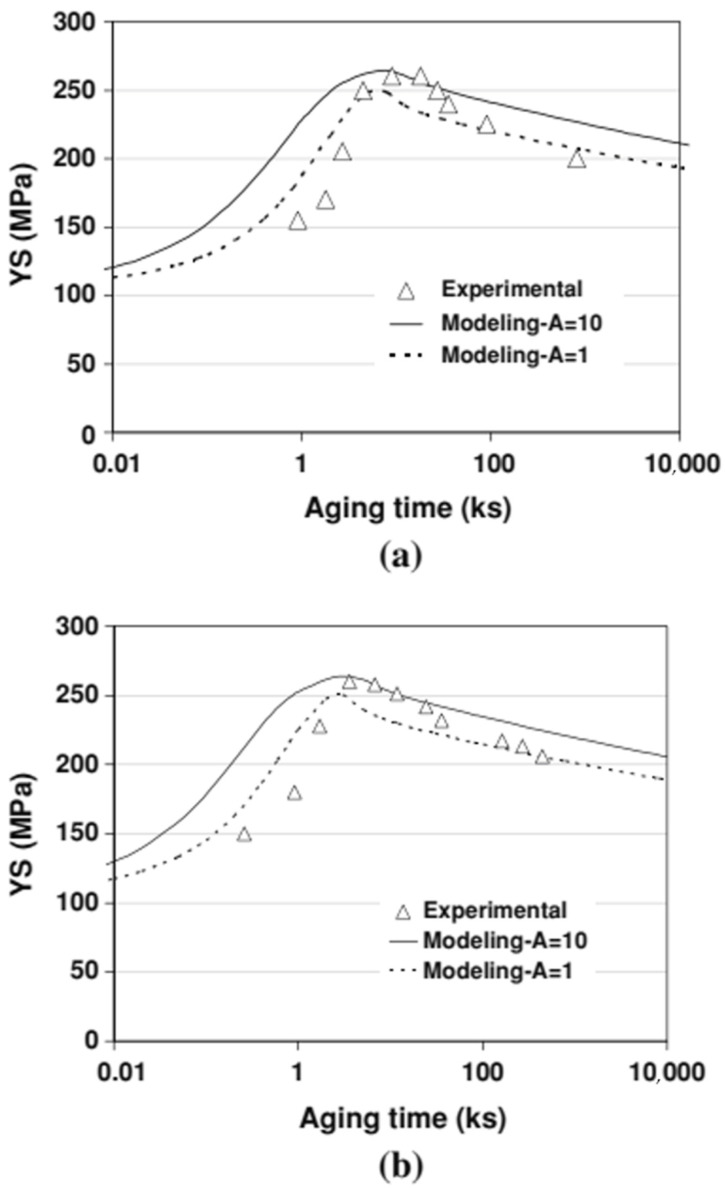
The aspect ratios A = 1 and 10 predict the yield strength of alloy AA 6061 aged at (**a**) 190 °C and (**b**) 205 °C [110].

**Figure 23:**
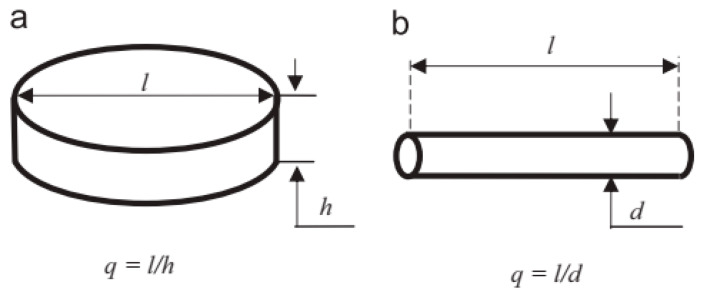
Morphology of (**a**) plate/disc or (**b**) rod/needle precipitates [111].

**Figure 24:**
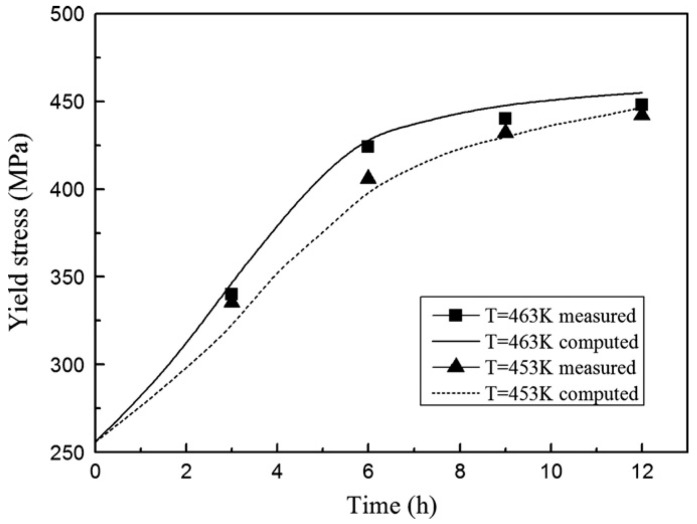
Measurement and prediction of yield stress changes in 2124 aluminum alloy aged at 453 K and 463 K [111].

**Figure 25:**
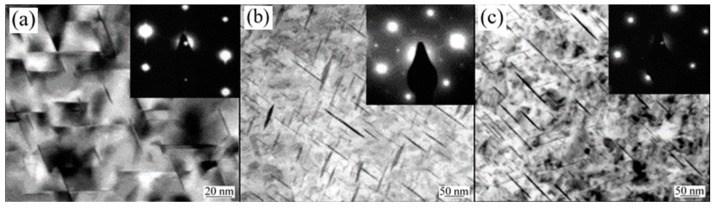
TEM images of alloy precipitates and corresponding diffraction points under different peak aging conditions: (**a**) 165 °C, 10 h; (**b**) 200 °C, 4 h; (**c**) 250 °C, 10 min [114].

**Figure 26:**
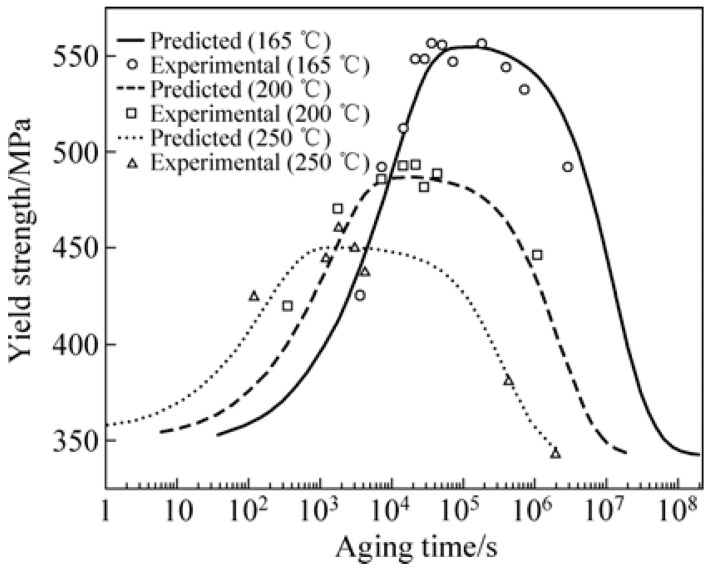
Experimental results and model predictions of yield strength of alloys aged at different temperatures [114].

Bahrami et al. [110] developed an age-hardening model for Al-Mg-Si alloys, assuming that precipitates are cylindrical with a constant aspect ratio. This model successfully predicted the yield strength evolution of Al-Mg-Si alloys during aging (Figure 22), demonstrating that the aspect ratio significantly influences strengthening effects. They also noted that improving prediction accuracy requires developing a model capable of simulating non-constant aspect ratios, which are influenced by precipitate–matrix interface energy and interfacial reaction kinetics. Zhang et al. [111] established a unified constitutive model for creep age forming of heat-treatable aluminum alloys containing plate- or rod-shaped precipitates (Figure 23). This model incorporates microstructural evolution parameters such as dislocation density, precipitate size, volume fraction, and aspect ratio, which are critical for creep-aging strengthening. The static aging component of the model aligns well with the observed characteristics of precipitation strengthening responses. Figure 24 shows the predicted and experimental values of 2124 aluminum alloy yield strength after aging at different temperatures. Myhr et al. [112] proposed a new model to describe the artificial aging behavior of Al-Mg-Si alloys after cold deformation and room-temperature storage. This model confirms the applicability of the simplified spherical precipitate assumption and uniformly describes solute partitioning and competition mechanisms for different hardening phases (e.g., clusters, β″, etc.) based on the Kampmann–Wagner formalism. Calibrated experimentally, the model demonstrates high prediction accuracy and is suitable for simulating thermomechanical processes such as plate forming, tensile bending, heat treatment, and welding of Al-Mg-Si alloys. Anjabin [113] extended the spherical precipitate model to non-spherical precipitates by introducing a new rate law to simulate the kinetics of non-spherical precipitate evolution during the aging process of aluminum alloys. Building on this, the age-hardening model successfully predicted the hardness evolution of Al-Mg-Si and AA6082 alloys. Hou et al. [114] developed a numerical model based on the Kampmann–Wagner framework to predict the microstructure evolution and yield strength of Al-Cu-Mg-Ag alloys throughout the aging process. The model integrates classical nucleation and growth theory with a thermodynamic precipitation model to track changes in precipitate radius, critical radius, and volume fraction at aging temperatures of 165 °C, 200 °C, and 250 °C. Figure 25 presents TEM images of the alloy precipitates and their corresponding diffraction points under different peak aging conditions. Additionally, Figure 26 demonstrates that the predicted yield strength values from the model align well with the experimental results. Bardel et al. [115] developed a coupled precipitation and strengthening model to describe the microstructure and strength evolution of alloy 6061 during non-isothermal treatments in the T6 state. The model incorporates fitted interfacial energy and solubility limit parameters and considers non-spherical obstacle distributions. It can predict microstructural inhomogeneities in the heat-affected zone of welds and their impact on mechanical properties. Mishra et al. [116] investigated the yield strength anisotropy of Al-Mg-Si alloys using plastic, modified plastic, and elastic inclusion models, introducing the effective Taylor factor (M_eff_) parameter. Their results show that the modified plastic inclusion model accurately predicts the trend and magnitude of yield strength variation with respect to the rolling direction angle. Additionally, the concept of the effective Taylor factor can be applied effectively without needing to account for the specific shape, orientation, or positive/negative contributions of precipitates to the matrix’s yield strength anisotropy. Seisenbacher et al. [117] proposed an improved physical aging model for simulating the evolution of material properties in Al-Si-Mg-Cu alloys at different aging and testing temperatures. The peak error between simulated and experimental results under the same temperature conditions was within ±2%.

Poole et al. [118] investigated the effect of pre-strain after solution treatment on the two-step artificial aging response of AA7030 and AA7108 alloys. They developed a predictive yield stress model based on an internal state-variable approach, incorporating factors such as the average spacing of precipitates, precipitate strength, and static recovery of the deformed structure. The model uses heat-treatment parameters and pre-strain levels as variables, and its predictions align well with experimental results. Ho et al. [47] developed physically based unified constitutive equations to describe age–creep behavior during industrial age forming of aluminum alloys. These equations can predict age hardening and creep deformations during and after age forming, as well as accurately simulate stress relaxation and springback in age-forming processes. Yazdanmehr et al. [119] developed a structural model to predict the precipitation hardening behavior of AA6061 alloy during non-isothermal aging before peak aging. By incorporating both precipitation and dissolution mechanisms, the model serves as a tool for predicting the evolution of mechanical properties in Al-Mg-Si alloys under non-isothermal heat-treatment conditions. Myhr et al. [120] introduced a bivariate internal state variable model to describe the interactions between solute atoms, precipitates, and dislocations, capturing the work-hardening behavior of commercial Al-Mg-Si alloys at room temperature. The study revealed that dislocation density can be decomposed into statistically stored dislocations and geometrically necessary dislocations. By converting dislocation density into equivalent flow stresses using dislocation theory, the model enables the evaluation of the effects of alloy composition, heat treatment, and welding on work hardening. Key microstructural parameters influencing room-temperature work-hardening behavior include the geometric slip distance and the volume fraction of Orowan particles, which can be extracted from the particle size distribution. Anjabin et al. [121] proposed a mathematical model to predict the flow behavior of age-hardened aluminum alloys under various heat-treatment and thermal deformation conditions. Based on the Kocks–Mecking–Bergström framework, the model incorporates the effects of solid solution time and temperature and introduces the relative volume fractions of precipitates into the flow stress calculations. The model’s predictions show excellent agreement with experimental results. Zhou et al. [122] developed a dynamic strain aging (DSA) kinetic model based on the relationships between strain rate, temperature, critical strain, and critical strain rate. Their results demonstrate that the competitive mechanism between dislocation stress and dynamic strain aging stress effectively explains the transition from decreasing to increasing critical strain. Lan et al. [123] calculated the precipitation kinetics of θ″, θ′, and θ phases in 2A14 aluminum alloy after solid solution treatment using the JMAK equation. They then established a strength prediction model following tensile and compressive deformation, investigating the effect of cold deformation before aging treatment on the alloy’s properties. Building on these findings, Wang et al. [124] developed a knowledge-based cloud–finite element simulation platform (KBC-FE). This platform integrates traditional finite element (FE) simulation, advanced physical model-based functional modules, and cloud computing to enable the design, development, and optimization of sheet metal forming processes. The platform was used to generate a post-forming strength optimization scheme by optimizing artificial aging conditions for the hot stamping of AA6082 aluminum alloy. The scheme demonstrated high prediction accuracy and can be utilized to customize post-forming performance through this platform.

For predicting the mechanical properties of aluminum alloys during hot stamping and age-hardening processes, numerous strength prediction models based on microstructural evolution have been developed. These models comprehensively consider precipitation kinetics, strengthening mechanisms, and their interactions with dislocations, covering the nucleation, growth, and coarsening of precipitates, as well as the effects of temperature, time, and deformation history on microstructure and yield strength evolution. However, challenges remain in improving model accuracy under complex processing conditions, such as simulating non-uniform precipitate morphologies, studying the coupling effects of dynamic strain and aging, and understanding property evolution under multiphysics interactions. Additionally, intelligent prediction platforms integrating cloud computing technology show significant potential in enabling the tailored design of aluminum alloy properties post-forming and optimizing industrial production processes with higher efficiency.

### 2.3. Optimization of HFQ^®^ Heat-Treatment Process

Strength prediction models provide a theoretical foundation for evaluating the performance of aluminum alloy formed parts. However, in practice, dynamic changes in the heat-treatment process such as rapid heating or cooling often cause deviations in material behavior from the ideal state. By analyzing key influencing factors in the model, heat-treatment steps can be optimized more accurately to achieve desired material properties [125,126].

#### 2.3.1. Rapid Solid Solution Technology

Heat-treatable aluminum alloys include 2XXX series aluminum alloys, 6XXX series aluminum alloys, and 7XXX series aluminum alloys. These alloys contain significant amounts of alloying elements such as copper (Cu), magnesium (Mg), silicon (Si), and zinc (Zn), which dissolve in aluminum. The solubility of these elements in aluminum varies significantly with temperature, being much higher at elevated temperatures than the equilibrium solid solubility at room or moderate temperatures, and may even exceed the maximum solubility at the eutectic temperature. During solid solution treatment, solute elements are fully dissolved into the aluminum matrix by holding at appropriate temperatures for an extended period, forming a single-phase α-solid solution [121,127]. This creates a supersaturated solid solution, providing an excellent foundation for subsequent quenching and aging treatments, thereby significantly enhancing mechanical properties. The key to effective solid solution treatment lies in precise control of temperature and holding time. Higher solution treatment temperatures accelerate the dissolution of strengthening phases; however, excessively high temperatures can cause grain coarsening or over-burning [128]. Conversely, temperatures that are too low may prevent the complete dissolution of strengthening phases, significantly reducing solute concentration and ultimately compromising strength and hardness. Therefore, an optimal solution treatment balances temperature and time to maximize the formation of a supersaturated solid solution while avoiding over-heating, over-burning, and excessive grain growth.

**Figure 27:**
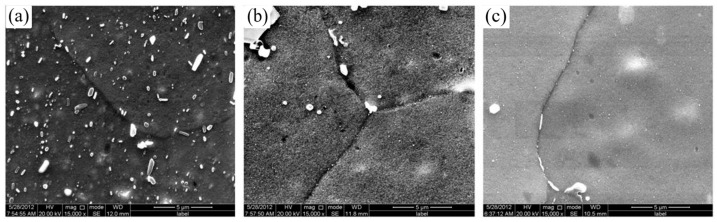
SEM images for different solution times: (**a**) 5 min, (**b**) 25 min, (**c**) 50 min [131].

**Figure 28:**
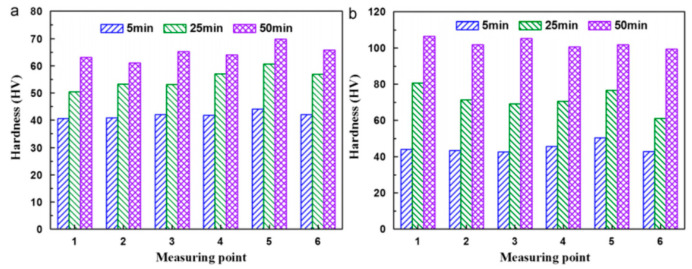
Hardness versus solid solution time: (**a**) quenched state, (**b**) aged state [131].

**Figure 29:**
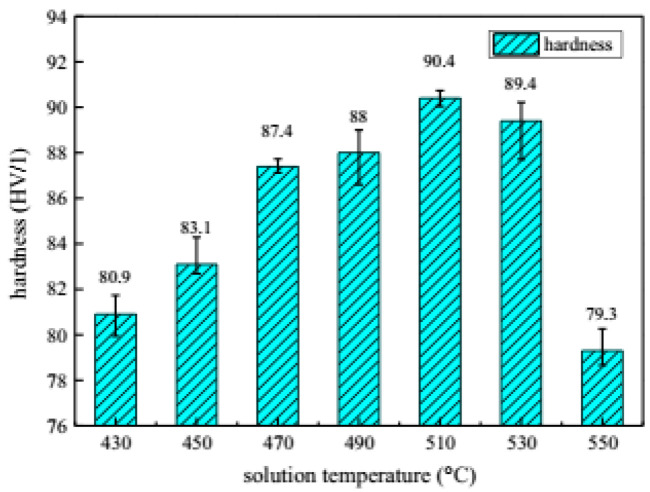
Solid solution temperature versus hardness [133].

**Figure 30:**
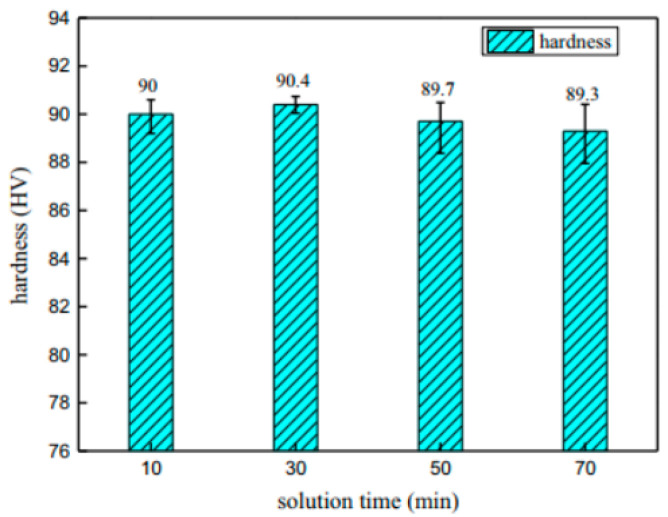
Solidification time versus hardness [133].

Garrett et al. [129] investigated the effect of solid solution time on the mechanical properties of AA6082 at different deformation rates. They found that increasing the proportion of solid solution time (defined as the duration at the solid solution temperature of 525 °C relative to the total solid solution time of 30 min) improves plasticity and reduces the maximum flow stress. When the solid solution time ratio reaches 0.66, further increases in solid solution time have minimal effects on the material’s mechanical properties, and initial anisotropy essentially disappears. Mrówka-Nowotnik et al. [130] studied the influence of solid solution heat-treatment temperature on the aging mechanism and kinetics of commercially deformed 6005 aluminum alloy. Their analysis revealed that the aging kinetics and hardness of 6005 aluminum alloy are generally independent of the solid solution heat-treatment temperature. Fan et al. [131] investigated the effect of different solid solution times (5 min, 25 min, and 50 min) on the hardness of a self-developed 6A02 aluminum alloy using the HFQ^®^ process in a specialized device. As shown in Figure 27, the solid solubility of the alloy increases with the length of dissolution time, SEM analysis reveals the time-dependent dissolution of secondary phase particles during solution treatment. Initially (5 min), particles remain largely undissolved, distributed unevenly in the Al matrix (Figure 27a). With longer treatment (up to 50 min), progressive dissolution occurs (Figure 27b,c), demonstrating increasing solid solubility over time in the quenched condition. Reaching its highest value after 50 min. Figure 28 illustrates that the alloy hardness increases with the solid solution time at a temperature of 520 °C, peaking at approximately 70 HV after 50 min of solid solution treatment. Following aging treatment at 160 °C for 10 h, the hardness further increased to 106.5 HV. Ma et al. [132] also focused on solid solution temperature and time as key design variables and developed a response surface model to predict the ultimate tensile stress and ductility of the alloy. Their findings revealed that ultimate tensile stress increases while ductility decreases with higher solid solution heat-treatment (SHT) temperatures and longer times. These parameters were later optimized using the multiobjective sorting genetic algorithm (NSGA-II) to enhance both tensile strength and ductility. Liu et al. [133] investigated the effect of different solid solution temperatures and times on the strength of 7075 aluminum alloy sheets under the HFQ^®^ process. They set the solid solution time to 30 min and observed changes in alloy hardness with varying solid solution temperatures. As shown in Figure 29, the maximum hardness of 90.4 HV was achieved at 510 °C. Subsequently, they compared the effect of different solid solution times on hardness at the optimal temperature of 510 °C, as illustrated in Figure 30. The results indicate that solid solution time has a less significant impact on hardness compared to temperature, with the peak hardness of 90.4 HV reached precisely at 30 min.

Precise control of the solid solution treatment regime is essential for achieving excellent mechanical properties in aluminum alloys. Optimizing solid solution temperature and time for different aluminum alloys is central to enhancing the efficiency of the HFQ^®^ forming process. However, in traditional aluminum alloy hot stamping, the solid solution treatment time is typically long, often around 30 min, primarily due to the low thermal radiation absorption of aluminum alloy sheets [134]. This prolongs the time required to reach the solid solution temperature. While increasing the solid solution temperature can shorten treatment time, it also raises the risk of over-burning [39]. Additionally, subsequent aging often takes several hours, further reducing productivity. Modern hot stamping lines operate at extremely fast paces (approximately 20 s per cycle), creating a significant mismatch between the stamping cycle and the required solution and aging times. This makes it challenging to maintain production continuity [135]. To address this issue, recent research has focused on shortening the cycle times for solution and aging processes. Scholars have explored optimizing heating methods and treatment parameters to achieve more efficient heat treatment of aluminum alloys, meeting the demands of industrial-scale hot stamping [136,137].

**Figure 31:**
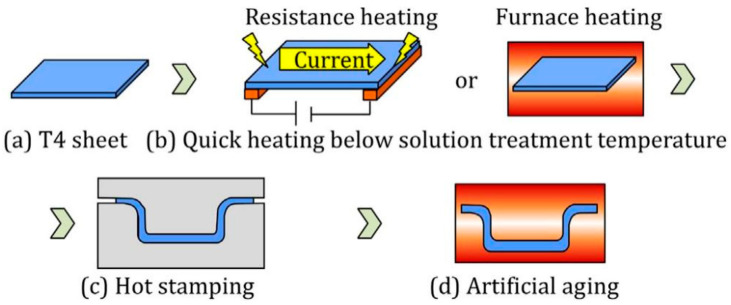
Hot stamping process for high-strength aluminum alloy aircraft parts using rapid heating [145].

**Figure 32:**
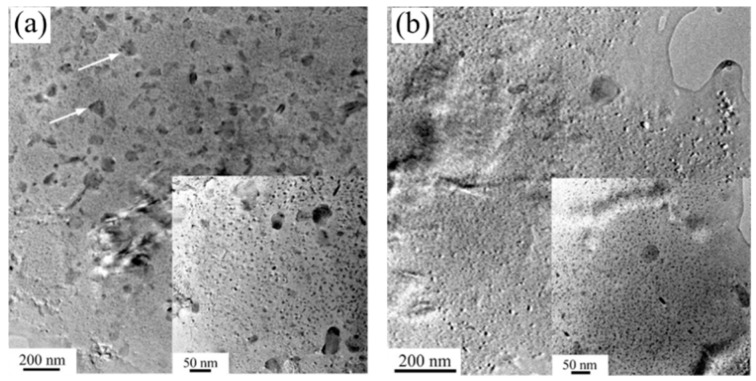
TEM micrographs of the aged 7075 Al alloy treated at (**a**) SST + AA and (**b**) EPT + AA [151].

The key to accelerating the solid solution treatment of aluminum alloys lies in increasing the heating rate. Rapid heating not only significantly reduces the solution time [138], but also helps prevent the formation of transient phases [139], which have minimal impact on the final mechanical properties of the part. One effective technique is conductive heating, where copper electrodes are clamped at both ends of the sheet, allowing a high-power current to flow through the heating area. This process generates substantial Joule heat, enabling rapid temperature rise [140,141,142,143,144]. Maeno et al. [145] compared the heating efficiency of resistance heating and radiant furnace heating for T4 aluminum alloy sheets (A2024 and A6061) in a hot stamping process, as illustrated in Figure 31. Their experiments demonstrated that resistance heating could achieve a solid solution temperature of 520 °C in just 3.4 s, whereas conventional radiant furnace heating required approximately 420 s. Additionally, formed parts processed via resistance heating exhibited lower surface roughness. However, due to uneven temperature distribution across the sheet, this method is primarily suitable for small, narrow, rectangular components [146]. For larger or irregularly shaped parts, hybrid heating strategies may be necessary to ensure uniform temperature distribution [147]. In recent years, electric pulse technology has been widely applied in the heat treatment of metals and alloys [61,148]. Xu et al. [149] conducted electric pulse treatment on cold-worked AZ31 alloy and found that it accelerated recrystallization and nucleation rates. Zheng et al. [150] investigated electric pulse solid solution treatment (EPST) for 6061 aluminum alloy. Compared to conventional solid solution treatment, EPST completed the dissolution process within 15 s, increasing the tensile strength of treated specimens by 6.8% and Vickers hardness by 7.2%. Xu et al. [151,152] applied electric pulse treatment to hot-rolled 2024 and 7075 aluminum alloys, observing that pulsed current treatment significantly accelerated the solid solution process of 7075 aluminum alloy, yielding similar conclusions. As shown in Figure 32, the dissolution degree of EPST is lower compared to conventional solid solution treatment. However, the strength of EPST-treated samples is slightly higher than that of SST-treated samples, with a minor reduction in plasticity. This can be attributed to the fine-grained structure induced by the electric pulse, which promotes solute homogenization and inhibits the coarsening of precipitated phases. Additionally, Liu et al. [153] integrated the resistance heating technique into the HFQ^®^ process by heating U-shaped 5A90 aluminum–lithium alloy parts using a high-density DC pulsed power supply. The results demonstrated excellent forming accuracy, achieving a minimal springback angle of just 0.43°.

**Figure 33:**
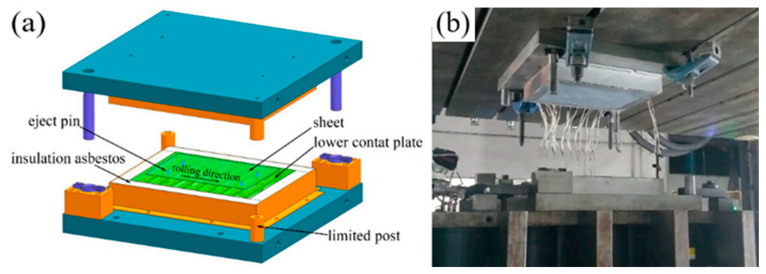
(**a**) Structural model of the contact heating device. (**b**) Actual structure of the contact heating device [154].

**Figure 34:**
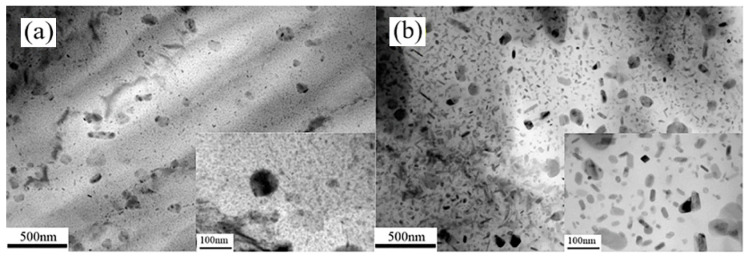
TEM micrographs of 7075 aluminum alloy after AA: (**a**) FST + AA, (**b**) CST + AA [157].

Geng et al. [154] designed a contact heating device (shown in Figure 33) to heat 7075 aluminum alloy plates. Contact heating utilizes direct heat conduction through the contact between a high-temperature plate and the material. In the experiment, the aluminum alloy plate reached a temperature close to the set value within 15–20 s, demonstrating significantly higher heating efficiency than conventional radiant heating. Furthermore, the heating time showed minimal dependence on the set temperature. The temperature distribution across the contact surface was also more uniform, with a minimum temperature difference of less than 20 °C. From a material properties perspective, contact heating effectively shortened the solid solution cycle. Zhang et al. [155,156,157,158] further investigated the contact solid solution treatment (CST) of 7075 aluminum alloy and found that the second-phase particles fully dissolved into the aluminum matrix within 40 s, while the peak aging time was reduced to 18 h. As shown in Figure 34, CST proved to be more efficient and effective than conventional furnace-based solid solution treatment. The precipitated phases in CST samples were significantly refined and more numerous, leading to slightly higher strength in the aged state compared to the conventional T6 condition. Additionally, the strength of 7075 aluminum alloy subjected to contact solid solution treatment increases with higher contact body temperatures and extended holding times. However, excessively high solid solution temperatures or prolonged holding times can negatively impact mechanical properties. Studies indicate that Orowan strengthening is the primary strengthening mechanism in the contact solid solution treatment of 7075 aluminum alloy. Rasera et al. [159] also investigated the contact heating process for high-strength steel billets. Using a SiC-based heating material, they heated the billet to the Ac3 temperature within 60 s, achieving an overall temperature of 1050 °C within 1 h. The study further revealed that the billet coating exhibited strong adhesion to the substrate throughout the heating process.

**Figure 35:**
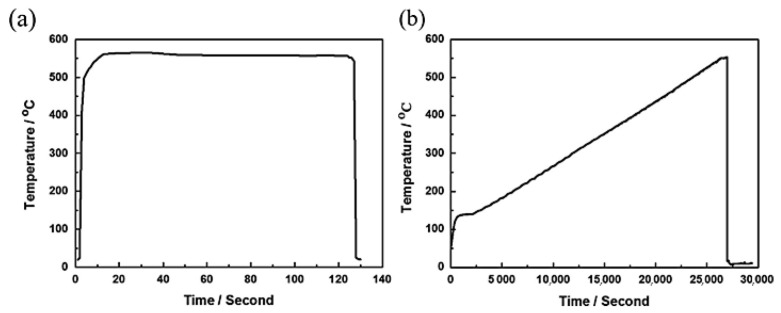
Solid solution treatment curves for different processes: (**a**) salt-bath furnace, (**b**) air furnace [160].

Wang et al. [160] investigated the solid solution treatment of Al-Mg-Si-Cu alloy plates using both salt-bath and air-furnace heating methods. Their results showed that the salt-bath heating achieved an average heating rate of 60 °C/s, whereas the air furnace exhibited a much slower rate of only 1 °C/min (Figure 35). Previous studies have indicated that solution treatment of 6082 aluminum alloy at 530–565 °C typically requires approximately 6 h to reach peak-aged tensile yield strength, resulting in an overall heat-treatment cycle lasting several hours [161,162]. Chang et al. [134] compared the effects of salt-bath heating and resistance-furnace heating on the solution treatment of A6082 at the same solid solution temperature (560 °C) and identical quenching conditions. Their findings demonstrated that salt-bath heating significantly reduces the treatment time while maintaining a finer grain size distribution, thereby ensuring the mechanical properties of the alloy.

**Figure 36:**
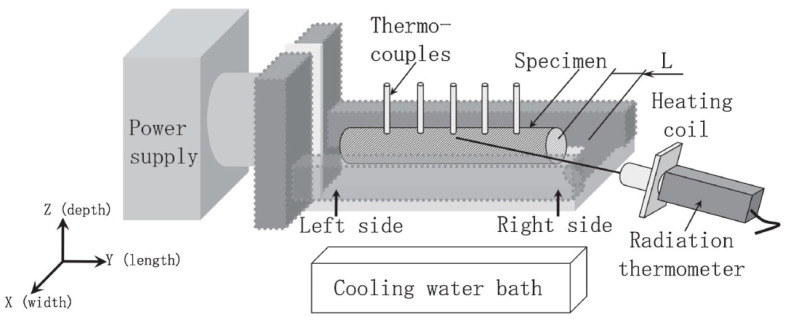
Schematic diagram of the experimental setup [165].

In recent years, induction heating has been widely applied to the heating of metal sheets, particularly high-strength steel plates. The induction heating system typically comprises a high-frequency generator and an induction coil. The temperature of the sheet is elevated through resistive heat generated by eddy currents induced in the metal by the coil [163]. Kolleck et al. [164] utilized an induction heating device to heat an uncoated 22MnB5 plate to 950 °C within 5–60 s. Similarly, Shang et al. [165] applied high-frequency induction heating to 6061 aluminum alloy bars, as illustrated in Figure 36. Their results revealed uniform heating with minimal temperature variations at a heating rate of 3.5 °C/s, achieving rapid linear heating rates of up to 21 °C/s. Moreover, the heating rate during the heat-treatment process exhibited negligible effects on the mechanical properties of the 6061 aluminum alloy, suggesting that induction heating significantly enhances solid solution efficiency. However, the efficiency of induction heating is influenced by several factors, including current frequency, sheet conductivity, magnetic permeability, and hysteresis loss in magnetic materials. Additionally, the current frequency determines the magnetic field penetration depth, which is greater at lower frequencies and smaller at higher frequencies.

**Figure 37:**
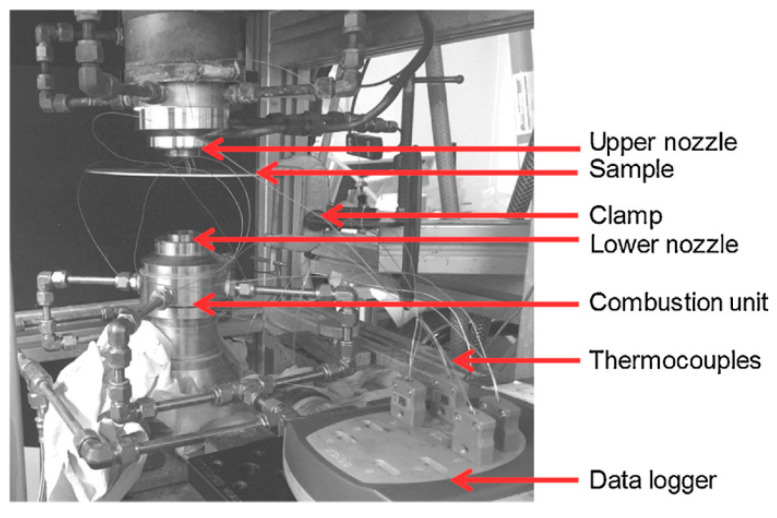
Setting up DFI heating in an open environment [166].

Shao et al. [166] proposed the direct flame impingement (DFI) heating method for experiments on AA6082 thin plates. The flame heating device, illustrated in Figure 37, achieved a heating rate exceeding 10 °C/s. This method demonstrated excellent surface layer chemistry and wettability under high heating rates and short holding times. Additionally, DFI effectively minimized grain growth during heating, proving to be a feasible, energy-efficient, and cost-effective approach. By integrating DFI with the HFQ^®^ process, it has the potential to significantly enhance productivity in industrial applications [167,168].

The emissivity (and absorptivity) of aluminum alloy surfaces was enhanced by Liu et al. [169] through the application of coatings such as boron nitride (BN). Experiments were conducted to measure the heating profiles of bare plates and those coated with BN or graphite in a radiant-heating furnace. It was found that the heating efficiency was increased by a factor of 7.4 for BN coatings and 9.2 for graphite coatings. By applying these coatings, the solid solution time for 6061 and 7075 aluminum alloys was reduced from 10 min to 5 min. In addition, the use of near-infrared heaters for rapid solid solution treatment of 7075-T6 aluminum alloy plates was investigated by Jung, Seon-Ho et al. [161]. A contact heater capable of achieving precise temperature control within a short time was developed. However, challenges such as low efficiency and uneven temperature distribution were observed for uncoated aluminum billets [170].

In summary, the regulation of the solid solution process for heat-treatable aluminum alloys (such as Al-Cu, Al-Mg-Si, and Al-Zn-Mg series) plays a crucial role in determining the mechanical properties of the alloy after forming. To achieve a supersaturated solid solution, the solid solution time must be carefully controlled while heating methods are optimized. Various heating techniques, including resistance heating, contact heating, salt-bath heating, induction heating, and flame heating, have been developed to enhance solid solution efficiency and optimize mechanical properties. Among these, resistance heating, contact heating, and coating technologies have demonstrated significant potential in reducing solid solution time. However, the process must be carefully managed to avoid issues such as over-cooking and grain coarsening.

#### 2.3.2. Rapid Aging Technology

For the large-scale application of the HFQ^®^ process in automotive aluminum alloy formed parts, the reduction in the heat-treatment cycle is recognized as a critical factor for improving productivity and cost-effectiveness [139]. While the solution treatment is time-consuming, the aging process occupies nearly the entire heat-treatment cycle. As previously described, significant advancements in rapid solution technology have been achieved, and the development and application of short-time aging are expected to further streamline the HFQ^®^ process.

The acceleration of the aging process has been explored through various approaches. Lin et al. [171] investigated the application of stress to accelerate aging, while Xu et al. [172] discovered that trace amounts of silver (Ag) significantly enhanced the precipitation process in 7075 aluminum alloy. Specifically, the addition of 0.4 wt% Ag was found to reduce the aging treatment time by 50% while improving the alloy’s strength and plasticity. This improvement is attributed to the formation of numerous Mg-Ag clusters [173]. To further accelerate the aging reaction, the heat-treatment history of the material should be fully utilized. Paint baking (PB), a necessary treatment for automotive body panels, is considered an effective method for achieving short-term strengthening of aluminum alloys [174,175]. Previously, numerous studies have focused on developing appropriate low-temperature pre-treatments before the paint baking (PB) process to enhance the baking response [176,177]. These short-duration artificial pre-treatments were designed to mitigate the effects of natural aging [178,179] and improve the PB response [180,181,182,183]. Li et al. [139] proposed a two-stage aging process combined with the baking process for AA6082 aluminum alloys. The optimal conditions were identified as 15 min of pre-heating followed by 220 °C × 5 min, which reduced the aging time by over 90% while maintaining strength compared to standard aging. Yin et al. [184] prepared Al-0.42Mg-0.5Si-1.0Cu alloys through hot extrusion, followed by quenching and aging. It was found that pre-straining before aging introduced dislocations, inhibited the formation of cluster GP zones, accelerated the nucleation of β″ and Q’ phases, and enhanced the alloy’s strengthening. Specifically, pre-stretching by 5% before aging increased the peak aging hardness of the alloys from 122 HV to 163 HV, while reducing the peak aging time to 2 h. Although pre-straining mitigates the effects of natural aging on the hardening behavior of the alloys, it promotes precipitate coarsening, leading to a significant decrease in hardness during the over-aging stage. Omer et al. [58] applied a primary aging treatment to AA7075 and AA7xxx alloys using a baking cycle of 177 °C × 30 min. This treatment enabled the alloys to achieve T6 or T76 properties while reducing the aging treatment time by 65–83% compared to standard T6 and T76 processes.

Zheng et al. [49] conducted an in-depth study on the rapid aging treatment of AA6082 aluminum alloy and established an optimized rapid aging process based on the HFQ^®^ process. The process consists of pre-aging (210 °C × 30–55 min) followed by baking (180 °C × 30 min). This approach was shown to maintain 95% of the alloy strength compared to standard aging while preserving 8% plasticity. Similarly, Jiang et al. [185] investigated two aging treatments for AA7075 within the HFQ^®^ framework: (i) 120 °C × 24 h and (ii) pre-aging (180 °C × 5 min) followed by simulated baking (180 °C × 30 min). Both treatments were found to result in satisfactory mechanical properties, but the pre-aging and simulated baking approach significantly reduced the overall processing time. Furthermore, Wang [186] developed the Rapid Light Alloy Stamping (RLAS) technology, which drastically shortened cycle times and has been successfully applied to AA6xxx aluminum alloys [125].

In summary, various rapid aging techniques have been explored, including electric-pulse aging treatment [187], cyclic strain at room temperature [188], and the addition of alloying elements to accelerate aging and enhance mechanical properties [189,190]. However, current research remains incomplete, and the available rapid aging methods are relatively limited. Among the existing approaches, pre-aging followed by baking has been demonstrated to be an effective strategy for reducing the aging cycle, particularly for certain high-strength aluminum alloys. Nevertheless, further research is required to develop rapid aging techniques that can be applied to a broader range of aluminum alloys used in the automotive and aerospace industries.

## 3. Aluminum Alloy Hot Stamping—New Process Research

The HFQ^®^ technology integrates the quenching process with forming to achieve superior formability, dimensional accuracy, and mechanical properties in the final parts. However, the HFQ^®^ process for aluminum alloys still faces several challenges, including the high cost associated with stamping die design and machining, significant wear on forming tools, and difficulties in shearing operations due to the high strength of the finished parts [191]. Notably, the extended duration of solution treatment and subsequent aging processes results in low productivity and high costs. Additionally, longer production cycles can lead to unstable product performance. As previously mentioned, researchers have successfully reduced the forming cycle time in hot stamping by optimizing heat treatment, particularly by shortening the solid solution and aging treatment times in the HFQ^®^ process. Meanwhile, alternative methods to reduce process cycle times are being explored by other scholars through innovative approaches.

### 3.1. Pre-Cooling HFQ^®^ Process

**Figure 38:**
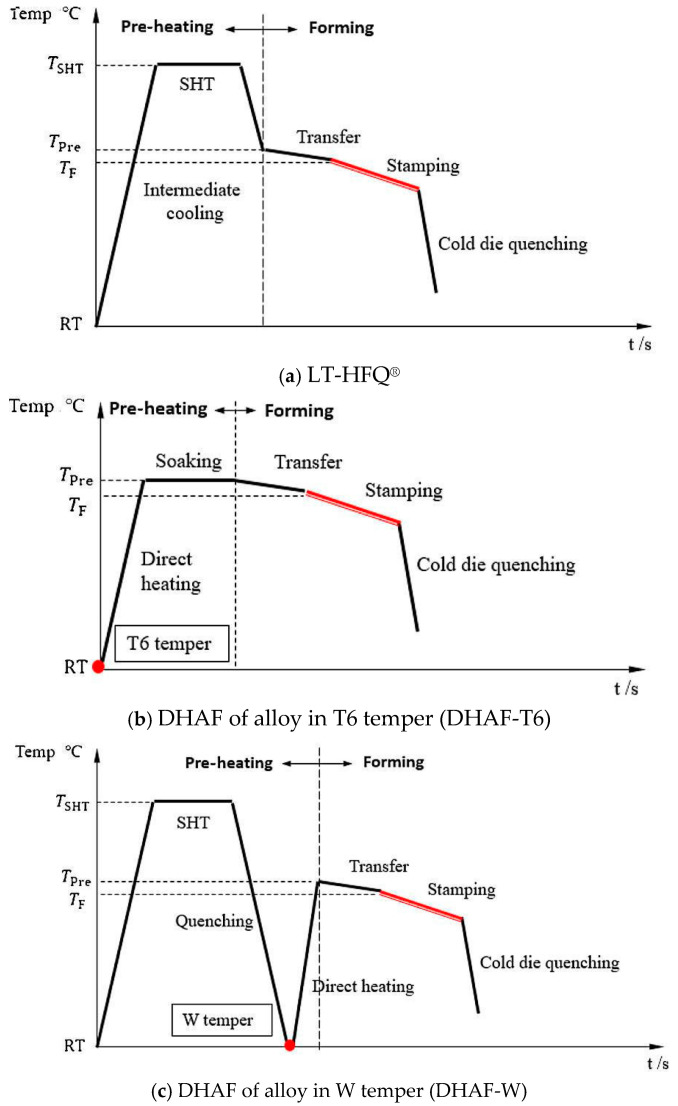
Temperature profiles for LT-HFQ^®^ and DHAF processes [196].

**Figure 39:**
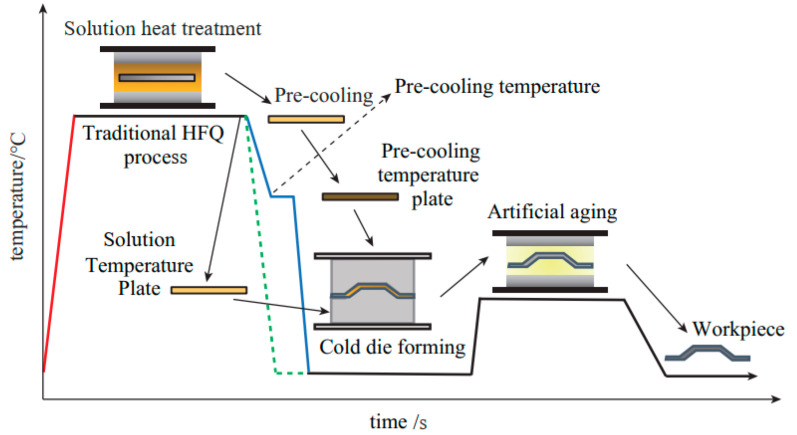
Comparison of the two process flows.

Wang et al. [75] demonstrated that the enrichment of solute atoms in AA2024 at the solid solution heat-treatment temperature leads to the softening of both the grain boundaries and the matrix, which reduces ductility and hampers forming capabilities. In the study of high-temperature formability of 7075-T6 aluminum alloy, it was found that the optimal deep drawing and tensile formability were achieved around 180 °C and 220 °C, respectively, which was significantly lower than the typical solution temperature [192]. To further optimize these properties, an enhancement to the HFQ^®^ process, known as LT-HFQ^®^ or pre-cooled HFQ^®^, was patented by Adam et al. [193] in 2015. This method was designed to improve forming at lower temperature ranges. Zheng et al. [194] simulated the in-die quenching process of hot-stamped aluminum alloy sheets at different temperatures. It was observed that quenching efficiency significantly improved as the initial die temperature decreased, validating the positive impact of LT-HFQ^®^ on enhancing quenching efficiency. Huo et al. [195] conducted warm uniaxial tensile tests and Erichsen tests on AA7075-T6 alloy, revealing that the material exhibited optimal formability at 200 °C. However, its strength was observed to decline significantly when formed at 250 °C. Zheng et al. [196] proposed an improved direct hot forming process (DHAF) and compared it with LT-HFQ^®^. The process temperature profile is illustrated in Figure 38. Under the same deformation conditions of 350 °C and 1 s^−1^, LT-HFQ^®^ was found to demonstrate significant strain hardening, improved ductility, and superior deep-drawing properties. These improvements are closely related to pre-heating conditions, and process variables such as temperature and forming speed were also shown to significantly influence the post-forming hardness of LT-HFQ^®^ deep-drawn parts. Further studies on hot deformation behavior and microstructure evolution revealed that the microstructure of highly quench-sensitive aluminum alloys is highly sensitive to temperature changes. For example, the precipitation of high-temperature S-phase (673–693 K) and intermediate-temperature phases (623–673 K) may deplete strengthening elements. Therefore, the hot stamping of high-strength, highly quench-sensitive aluminum alloys under pre-cooling conditions requires strict control of process variables, particularly pre-cooling conditions. In the pre-cooling HFQ^®^ process for 7075 aluminum alloy, the process is compared with conventional hot stamping, as illustrated in Figure 39. Zhu et al. [197] implemented two types of pre-cooling for alloy billets: air-cooled pre-cooling (ACPC) and cold-iron pre-cooling (CIPC). It was found that the formed parts exhibited minimal thinning at a CIPC pre-cooling temperature of 300 °C. Compared to ACPC, CIPC resulted in less precipitation during quenching and achieved the best mechanical properties after aging. Additionally, Li et al. [198] investigated the effect of hot forming on AA7075 aluminum alloy under pre-cooling conditions and developed a macro–micro coupled material model. Their study demonstrated that a lower pre-cooling rate (5 °C/s) leads to coarse precipitates, which negatively impacts formability. However, this research has yet to be validated on part-level workpieces.

### 3.2. High-Efficiency Hot Stamping Technology for Pre-Strengthened Aluminum Alloys

**Figure 40:**
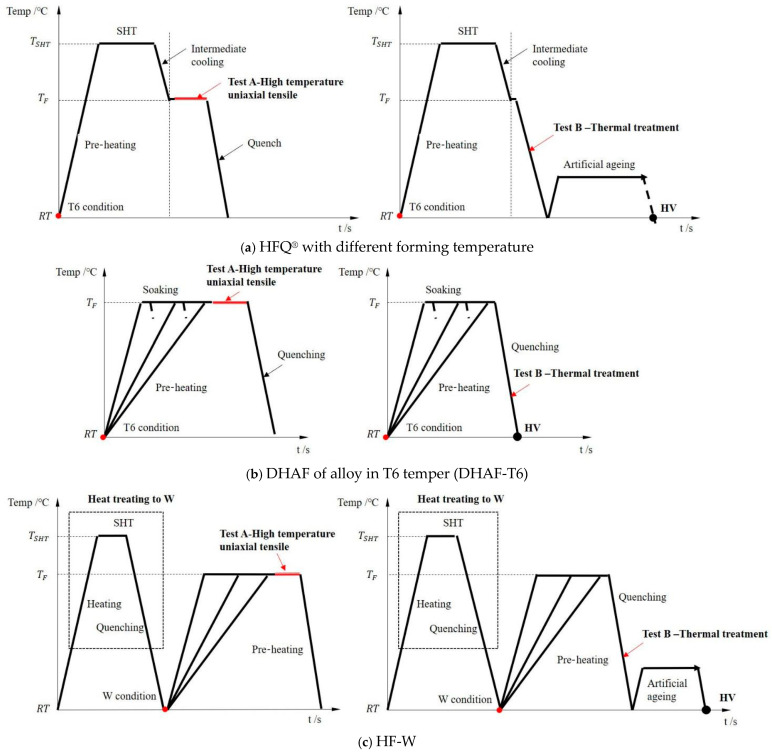
Schematic representation of different aluminum high-temperature forming processes: (**a**) HFQ^®^ with different forming temperatures, (**b**) HF-T6, and (**c**) HF-W [199].

**Figure 41:**
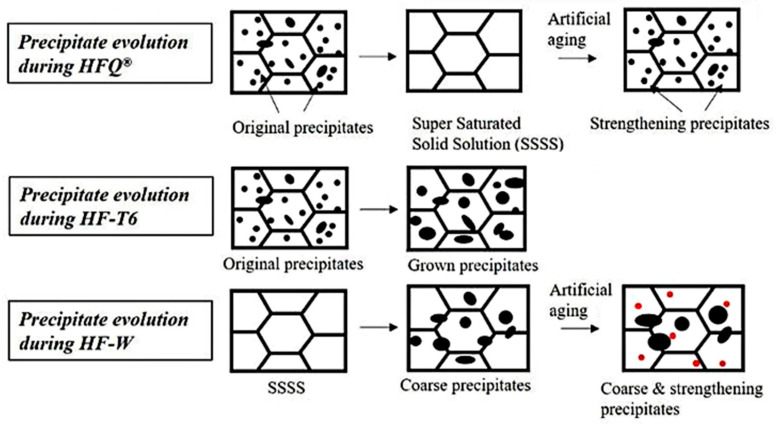
Microstructure evolution of aluminum alloys under different forming processes [199].

**Figure 42:**
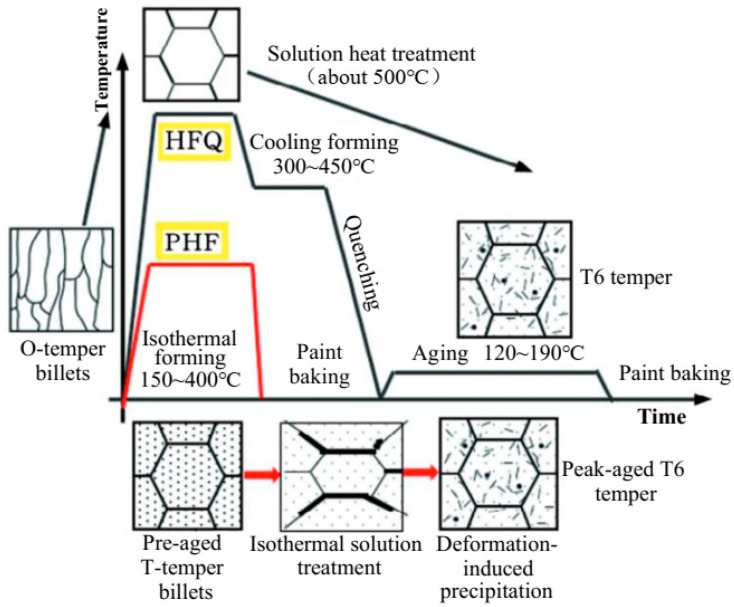
Comparison of the tissue evolution of high-efficiency hot stamping technique and HFQ^®^.

**Figure 43:**
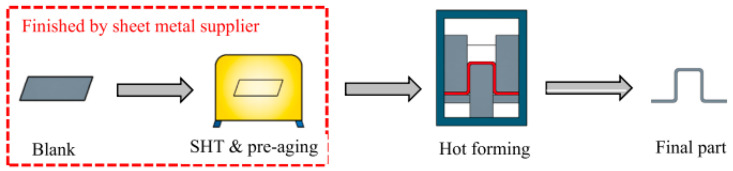
Schematic diagram of the PHF process chain [201].

The pre-cooled HFQ^®^ process significantly improves the forming accuracy of complex components through a gradient temperature control mechanism. However, its multistage process leads to exponentially longer cycle times, creating efficiency bottlenecks, particularly in the continuous production of large components. While the good ductility of heat-treatable aluminum alloys at low temperatures and the advantages of HFQ^®^ have been well established, few studies have focused on the direct forming of age-strengthened high-strength aluminum alloys. In this regard, Zheng et al. [199] investigated the forming performance of 7075 aluminum alloy under the direct hot forming (HF) process and the HFQ^®^ process. These processes were controlled through hot stretching and heat-treatment experiments, with various process variables being considered. The different high-temperature forming processes for aluminum alloys are illustrated in Figure 40. The results indicate that the hardness of optimally formed workpieces from the HF-T6 and HF-W processes is lower than that for workpieces formed using the HFQ^®^ process. This difference is attributed to the generation and growth of non-strengthening precipitates during pre-heating and high-temperature forming. The microstructural evolution of aluminum alloys under different forming processes is illustrated in Figure 41. For the HF process, the temperature of 7075 aluminum alloy near the nose of the TTP (time–temperature–property) curve was found to lead to a significant reduction in hardness. Therefore, process variables play a crucial role in tissue evolution during forming, resulting in changes in hardness. By combining tissue property regulation models and theoretical methods, Hua et al. [200] developed the PHF technology for aluminum alloys. This method replaces traditional soft-state aluminum alloy (O state) with pre-aged and strengthened aluminum alloy billets for direct hot stamping. Through the adjustment of phase transformation during the hot stamping and deformation process, under-aged billets are transformed into peak-aged components, enabling precise control over the strength and toughness of the formed parts. The principles of this method, compared to traditional hot stamping processes, and its workflow are illustrated in Figure 42 and Figure 43. In this process, the sheet, initially in the T6 state, undergoes a short heat treatment below the solid solution temperature (within a wide temperature range) in a furnace. It is then quickly transferred to a water-cooled die for stamping. The die remains closed to maintain pressure, and the shaped part is obtained immediately without requiring subsequent heat treatment. This method, which utilizes pre-aged blanks supplied in bulk by sheet metal suppliers, reduces solid solution heating time. The stamping process can be completed in minutes, eliminating the need for prolonged artificial aging, thereby significantly shortening the production cycle and reducing costs. Additionally, the T6 state aluminum alloy sheet, after a brief low-temperature heating, dissolves fewer precipitates into the matrix. The integrated stamping process leverages work hardening to ensure the strength of the formed sheet.

**Figure 44:**
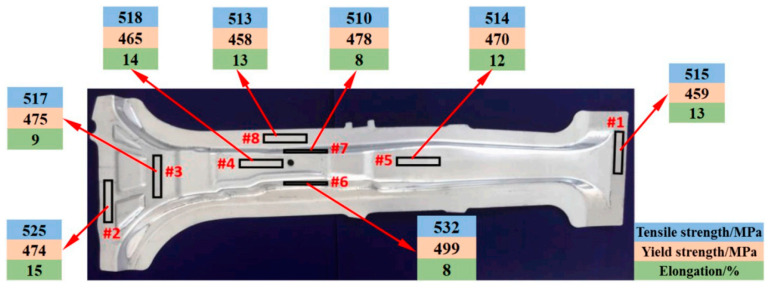
Distribution of mechanical properties of hot stamped parts at 200 °C [200].

**Figure 45:**
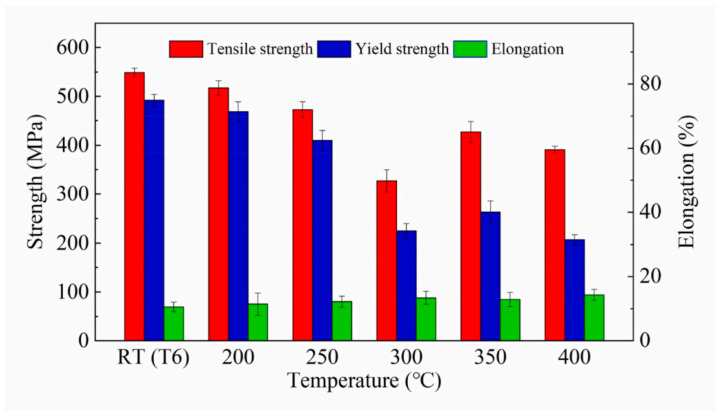
Mechanical properties of T6 alloy plates and hot stampings at different temperatures [200].

**Figure 46:**
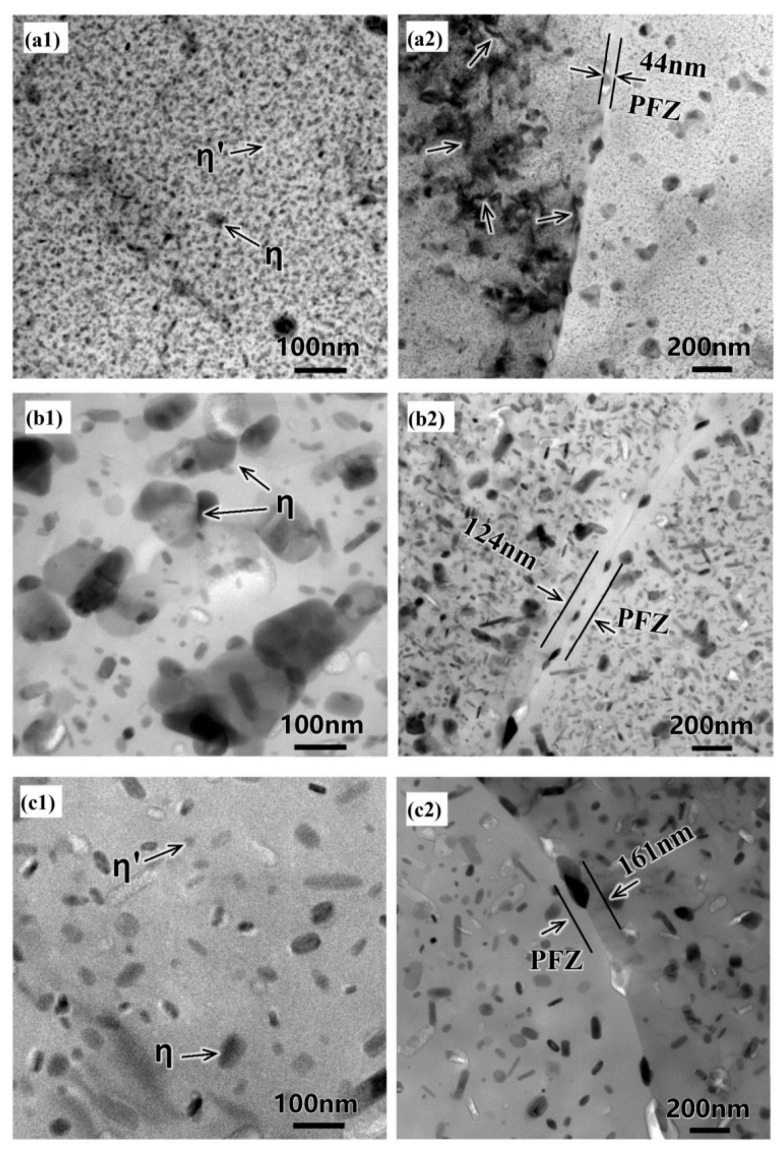
TEM images of AA7075 samples: (**a1**,**a2**) samples formed at 250 °C, (**b1**,**b2**) samples formed at 300 °C, (**c1**,**c2**) samples formed at 350 °C [200].

**Figure 47:**
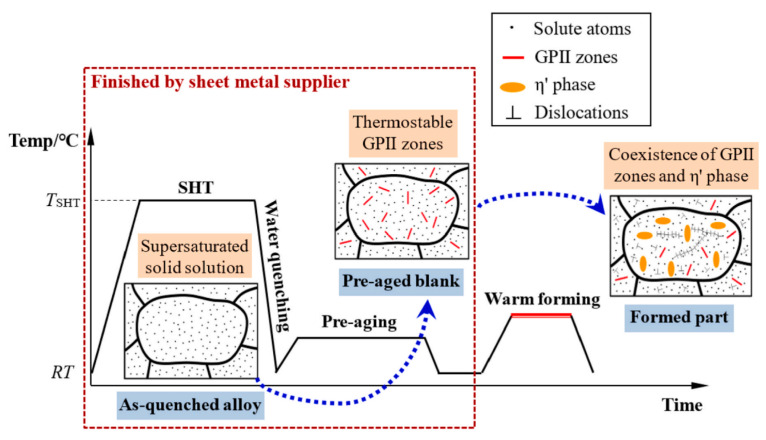
Schematic temperature distribution and microstructure evolution of the PHF process [201].

Zhang et al. [200] conducted hot tensile tests on AA7075-T6 alloy under the PHF process and observed that the alloy exhibited an elongation of 21% at a forming temperature of 200 °C. In automotive B-pillar stamping tests conducted at the same temperature, the mechanical properties of the formed parts were compared to those of the original T6 slabs, as illustrated in Figure 44 and Figure 45. The tensile and yield strengths of the formed parts were maintained at 92–96% and 93–97%, respectively, of the original T6 state blanks, with a minimum elongation of 8%. Additionally, specimens formed at 250 °C during deformation were found to contain a large number of η′ phases and dislocations, while the primary precipitated phase at 300 °C was identified as the coarse η phase, leading to a more significant strength drop (Figure 46). These findings indicate that direct forming of high-strength AA7075-T6 alloy can be achieved via the PHF process, offering a new approach for the forming of heat-treatable aluminum alloys. Subsequently, Hua et al. [201] investigated the effects of different pre-treatment and molding parameters on the PHF process of Al-Zn-Mg-Cu alloys. It was found that pre-aging treatment and solid solution time significantly influenced the properties of the stamped parts. Components with 12 h of pre-aging and 5 min of solid solution were observed to exhibit the best post-forming and baking strength. The study also revealed that the microstructural evolution during the PHF process primarily involves the transformation from the GPII region to the η′ phase, as illustrated in Figure 47. This transformation is accompanied by a large number of entangled dislocations and the pinning effect of precipitation on dislocations, which contribute to the high strength of the stamped parts. It is important to emphasize that the stamping process induces a strengthening mechanism rather than a softening one. Furthermore, the study suggests that the PHF process is applicable to other heat-treatable aluminum alloys, such as AA2xxx, AA6xxx, and AA7xxx.

**Figure 48:**
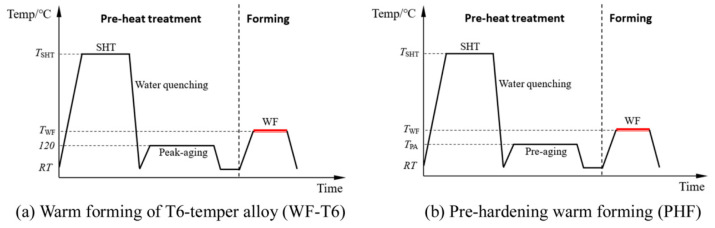
Temperature distribution for different molding processes: (**a**) WF-T6, (**b**) PHF [202].

**Figure 49:**
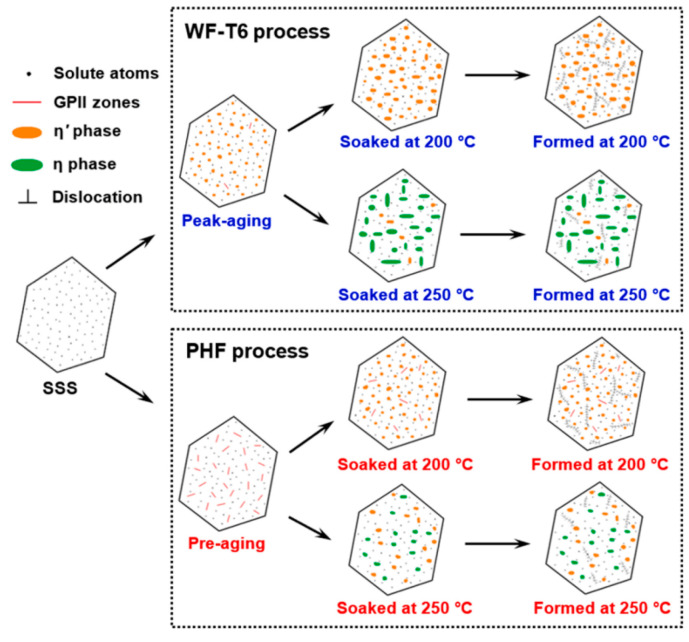
Microstructure evolution during WF-T6 and PHF processes [202].

**Figure 50:**
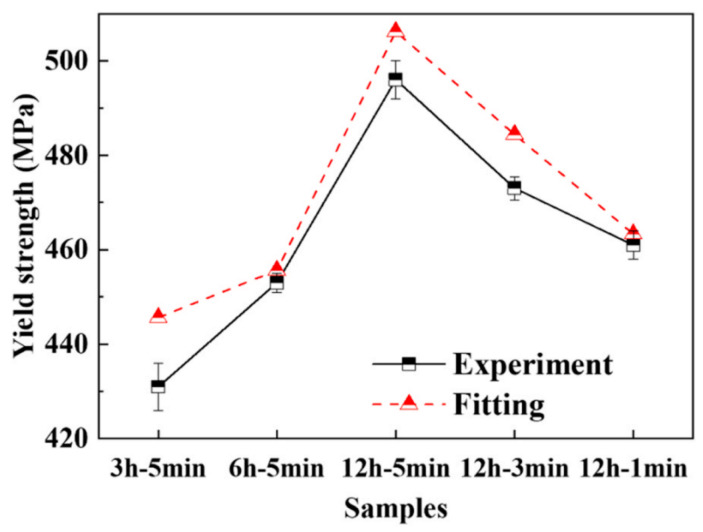
Comparison of experimental and modeled results for yield strength with different pre-aging times and solid solution times [203].

Based on the above work, Zhang et al. [202] compared the conventional T6 tempered alloy hot forming process (WF-T6) with the newly defined PHF for Al-Zn-Mg-Cu alloys, as illustrated in Figure 48. The study focused on forming performance at 200 °C. The results indicate that the PHF process provides better uniform elongation, tensile properties, and higher hardness compared to T6 sheets. The optimal PHF forming parameters were determined to be around 200 °C for 5 min. Additionally, the study highlighted significant differences in the degree of strength loss induced at 250 °C by different processes, as shown in Figure 49. Furthermore, the precipitation and strengthening behaviors in the pre-hardening forming of high-strength Al-Zn-Mg-Cu alloys were investigated by varying pre-aging times and solid solution times. It was found that the strength of parts formed using the PHF process (475 °C/30 min + 8% deformation) after pre-treatment (solid solution treatment at 475 °C/30 min + pre-aging at 85 °C/12 h) was comparable to that of T6 alloy. Although the volume fraction of the η′ phase in the formed parts was lower than that in the T6 alloy, this deficiency was offset by the GPII zones, which provided a strength contribution comparable to that of the η′ phase. Based on this finding, a yield strength prediction model incorporating the GPII zones and η′ phase was developed. The predicted values were found to deviate from experimental values by less than 5%, as shown in Figure 50 [203]. Hu et al. [204] determined the optimal process parameters for Al-Zn-Mg-Cu alloys under the PHF process: a pre-hardening temperature of 105 °C for 12 h, a deformation temperature of 190 °C for 5 min, and a strain rate of 1/s. The specimens were compressed to very high levels, demonstrating excellent ductility comparable to that of conventional forging processes. The study highlighted that the strengthening mechanism of the PHF process involves deformation-induced dynamic precipitation and the dual action of classical discontinuous precipitation. Additionally, Tang et al. [205] investigated the effect of different natural aging times (ranging from 48 h to 1 month) on the alloy before and after pre-hardened hot forming, examining its influence on the pre-hardened forming process.

The primary advantage of LT-HFQ^®^, as a modification of the HFQ^®^ process, lies in its ability to enhance the formability of aluminum alloys by incorporating a pre-cooling stage before forming and quenching. This approach improves the ductility and deep-drawing capabilities of aluminum alloys while mitigating material softening at elevated temperatures. Additionally, LT-HFQ^®^ significantly increases the efficiency of in-die quenching by reducing the overall process temperature. Furthermore, it minimizes the formation of precipitates in highly quench-sensitive aluminum alloys, preventing the depletion of strengthening elements and thereby preserving the material’s mechanical properties. However, LT-HFQ^®^ also presents challenges, particularly in process control. For highly quench-sensitive alloys, variations in pre-cooling conditions can lead to significant microstructural inconsistencies, resulting in the formation of coarse precipitates and unstable mechanical properties. From an industrial standpoint, LT-HFQ^®^ introduces an additional pre-cooling step to the HFQ^®^ process, primarily enhancing quenching efficiency. However, its impact on production time and cost remains comparable to that of the conventional HFQ^®^ process. Moreover, the comprehensive evaluation of LT-HFQ^®^ performance is still limited, and its actual industrial benefits require further investigation. The pre-aged hot forming (PHF) process represents advancement over conventional HFQ^®^ technology. Its key innovation lies in utilizing pre-aged aluminum alloy blanks, which eliminates the need for prolonged solution heat treatment and artificial aging steps required in HFQ^®^. This modification substantially reduces cycle times while maintaining satisfactory strength levels in formed components. The PHF process involves only brief low-temperature heating, hot press forming, edge trimming, and a short baking treatment—typically completed within minutes—offering significant time savings. Compared to HFQ^®^, the PHF process provides enhanced control over material toughness during forming and enables better property tuning in subsequent processing stages. However, current research on PHF reveals an inherent trade-off between the high-temperature formability of aluminum alloys with varying tensile strengths and the final mechanical properties of formed parts. From an industrial perspective, while the requirement for pre-aged aluminum blanks may increase material costs, the PHF process demonstrates considerable potential for future applications in aluminum alloy hot stamping technology.

## 4. Conclusions and Outlook

In recent years, significant breakthroughs have been achieved in the research of aluminum alloy hot stamping technology, particularly with the development of HFQ^®^ hot stamping technology. This innovation addresses long-standing challenges such as poor formability and severe springback of aluminum alloys at room temperature, establishing the way for large-scale industrial applications. This paper systematically summarizes the forming process parameters of HFQ^®^ technology, the thermal deformation behavior of aluminum alloys during the process, the material models, and optimization strategies. Research on forming process parameters reveals the complex effects of key factors—such as temperature, friction, speed, and crimping force—on forming performance, springback behavior, and thickness uniformity. These findings provide a theoretical foundation for the production of high-precision formed parts. The study of thermal deformation behavior and constitutive modeling has clarified the plastic deformation behavior of aluminum alloys at high temperatures, leading to the development of constitutive equations that describe the evolution of flow stresses and damage. A strength prediction model, which incorporates grain size effects and strengthening mechanisms, has been developed to further enhance the control over the mechanical properties of the formed parts. To address the limitations of the traditional HFQ^®^ process, improvements have been implemented through the use of resistance heating and contact solidification to effectively shorten the alloy’s solidification time, secondary aging during the aging process, and the application of baking varnish. These innovations help ensure the strength of the molded parts while optimizing the aging cycle. In addition, new processes for aluminum alloy hot stamping are currently in the exploratory stage. Recent studies, including LT-HFQ^®^ and PHF, have demonstrated the potential to improve productivity and mechanical properties. LT-HFQ^®^ optimizes process performance by introducing a pre-cooling stage, designing better forming temperatures for the sheet, and enhancing quenching efficiency. Meanwhile, PHF significantly reduces heating time and eliminates the need for prolonged aging while achieving high strength and quality in the formed parts. Overall, research on HFQ^®^ technology covering forming processes, material modeling, and especially heat-treatment optimization still requires further development. Based on this, studies on new processes such as LT-HFQ^®^ and PHF remain relatively limited, necessitating deeper exploration of alloy microstructure evolution and property modulation during these processes. At this stage, based on the above review and summary, significant work remains to advance these technologies:

The HFQ^®^ process involves numerous parameters that influence forming properties, such as deformation rate, die temperature, crimping force, and friction coefficient. However, current guidance on forming parameters for different aluminum alloys has not been systematically organized. It is essential to develop an integrated forming system to ensure optimal mechanical properties. Furthermore, the high manufacturing cost of stamping dies necessitates the development of precise control methods for alloy forming and cooling temperature fields. The integration of metal additive manufacturing technology into die production and defect repair offers significant advantages, enabling both rapid manufacturing and maintenance of hot stamping dies. Research into the wear mechanisms and lubrication methods between formed parts and dies is also crucial to effectively extend die life and improve the surface quality of formed parts.

The evolution of the microstructure of aluminum alloys during thermal deformation is highly complex. To accurately predict the deformation behavior and microstructure evolution of the material during the process, it is necessary to establish a multifactor coupled material model. Furthermore, the development of a high-precision strength prediction model for formed parts by integrating microstructural information and strengthening mechanisms will help reduce experimental costs.

From the perspective of the HFQ^®^ process cycle, research on rapid solution treatment and aging technologies still faces certain limitations. For rapid solution treatment, efforts are focused on developing fast and uniform heating methods, designing highly versatile equipment, and effectively preventing sheet over-burning. Regarding the optimization of the aging stage, studies on multistage aging systems that incorporate baking can significantly enhance the aging kinetics of aluminum alloys, enabling rapid precipitation strengthening through precise regulation of microalloying elements (e.g., Ag, Sc, Zr, etc.). Additionally, an in-depth investigation into phase transformation mechanisms under multifield coupling conditions, as well as the exploration of novel heat-treatment strategies—such as pulsed current-assisted treatment—could potentially break through the performance limitations of conventional processes. Simultaneously, the design and study of integrated hot stamping and forming equipment are essential to shorten the transfer time between various stages and further improve production efficiency.

The PHF hot forming process effectively addresses issues such as long cycle times and production beat mismatches in hot stamping. However, the influence mechanisms of pre-aging time and heating time on the mechanical properties of formed parts still require in-depth study. Meanwhile, the LT-HFQ^®^ process has shown promise in improving the overall quality of formed parts. Optimizing the parameters and workflows of PHF and LT-HFQ^®^ processes, as well as exploring new pre-strengthening methods, represent key future development trends.

In the future, there is still significant potential for the development of new hot stamping technologies beyond the LT-HFQ^®^ and PHF processes. Collaborative research between academia and industry will be crucial in addressing practical challenges and fostering innovation for high-performance applications. The emergence of artificial intelligence (AI) presents transformative opportunities for advancing HFQ^®^ and next-generation heat-treatment technologies. By leveraging machine learning and big data analytics, AI enables the digitization and automation of the entire manufacturing process, facilitating the transition from conventional methods to intelligent production systems. This integration can significantly enhance part quality, process efficiency, and overall manufacturing precision while reducing variability and production costs.

## Figures and Tables

**Table 1:** Comparisons of springback angles between simulation and experimental results [52].

Temperature/ °C	Springback Angle/(°)		Relative Error/%
	Experimental Result	Simulation Result	
**200**	***α* = 2.474**	***α* = 2.375**	**4.0**
**400**	***α* = 0.197**	***α* = 0.212**	**7.6**
**200**	***β* = 3.315**	***β* = 2.881**	**13.1**
**400**	***β* = 0.629**	***β* = 0.536**	**14.8**
